# Mosaics, mixtures, rotations or pyramiding: What is the optimal strategy to deploy major gene resistance?

**DOI:** 10.1111/eva.12681

**Published:** 2018-09-17

**Authors:** Loup Rimbaud, Julien Papaïx, Luke G. Barrett, Jeremy J. Burdon, Peter H. Thrall

**Affiliations:** ^1^ CSIRO Agriculture and Food Canberra Australian Capital Territory Australia; ^2^ BioSP, INRA Avignon France

**Keywords:** demogenetic model, deployment strategy, durable resistance, evolutionary epidemiology, gene‐for‐gene resistance, major gene resistance, *Puccinia*, spatially explicit modelling

## Abstract

Once deployed uniformly in the field, genetically controlled plant resistance is often quickly overcome by pathogens, resulting in dramatic losses. Several strategies have been proposed to constrain the evolutionary potential of pathogens and thus increase resistance durability. These strategies can be classified into four categories, depending on whether resistance sources are varied across time (rotations) or combined in space in the same cultivar (pyramiding), in different cultivars within a field (cultivar mixtures) or among fields (mosaics). Despite their potential to differentially affect both pathogen epidemiology and evolution, to date the four categories of deployment strategies have never been directly compared together within a single theoretical or experimental framework, with regard to efficiency (ability to reduce disease impact) and durability (ability to limit pathogen evolution and delay resistance breakdown). Here, we used a spatially explicit stochastic demogenetic model, implemented in the R package *landsepi*, to assess the epidemiological and evolutionary outcomes of these deployment strategies when two major resistance genes are present. We varied parameters related to pathogen evolutionary potential (mutation probability and associated fitness costs) and landscape organization (mostly the relative proportion of each cultivar in the landscape and levels of spatial or temporal aggregation). Our results, broadly focused on qualitative resistance to rust fungi of cereal crops, show that evolutionary and epidemiological control are not necessarily correlated and that no deployment strategy is universally optimal. Pyramiding two major genes offered the highest durability, but at high mutation probabilities, mosaics, mixtures and rotations can perform better in delaying the establishment of a universally infective superpathogen. All strategies offered the same short‐term epidemiological control, whereas rotations provided the best long‐term option, after all sources of resistance had broken down. This study also highlights the significant impact of landscape organization and pathogen evolutionary ability in considering the optimal design of a deployment strategy.

## 1 INTRODUCTION

In plants, genetically controlled qualitative (or “major gene”) resistance against a given pathogen is often described as providing complete (or at least strong) protection from infection (Parlevliet, 2002; Stuthman, Leonard, & Miller‐Garvin, 2007). However, once deployed in the field, such resistance has often been quickly overcome, resulting in dramatic epidemics and the need to identify and develop new sources of genetic resistance (García‐Arenal & McDonald, 2003; Johnson, 1984; Lecoq, Moury, Desbiez, Palloix, & Pitrat, 2004; McDonald & Linde, 2002; Parlevliet, 2002). Importantly, novel sources of resistance are not inexhaustible. Thus, several strategies have been proposed to improve major gene resistance durability. These strategies rely on the introduction of spatiotemporal variation in resistance in cultivated agroecosystems (Zhan, Thrall, Papaïx, Xie, & Burdon, 2015) and can be classified into four main deployment categories: (a) crop rotations, for example recurring succession of different crop cultivars in the same field (Curl, 1963); (b) mosaics, that is different cultivars in different fields of a continuous landscape (Burdon, Barrett, Rebetzke, & Thrall, 2014; Zhan et al., 2015); (c) mixtures, that is different cultivars combined in the same field (Mundt, 2002; Wolfe, 1985); and (d) pyramiding, that is different resistance sources stacked in the same cultivar (Ellis, Lagudah, Spielmeyer, & Dodds, 2014; Fuchs, 2017). At landscape scales, in addition to the possibility of combining several of these categories into more complex strategies, there are a diversity of deployment options within a category (e.g., choice of resistance sources, relative proportion and location of different cultivars in the landscape). Furthermore, as genetic engineering and gene editing technologies become increasingly powerful (e.g., CRISPR/Cas9), some strategies are now becoming more feasible (e.g., resistance mixtures composing isogenic lines with uniform phenologies and yield characteristics) (Koller, Brunner, Herren, Hurni, & Keller, 2018; Wang et al., 2014).

Given this diversity of options, identifying an optimal deployment strategy in a given epidemiological context is a challenge. Moreover, the criteria used to determine an optimal strategy depend on the objectives of a given stakeholder group (e.g., breeders, growers, risk managers) (van den Bosch & Gilligan, 2003; Papaïx, Rimbaud, Burdon, Zhan, & Thrall, 2018), noting that resistance durability (defined here as the ability to limit pathogen evolution and delay resistance breakdown, after which resistance is considered overcome) and epidemiological efficiency (defined as the ability to reduce disease impact or severity, as a result of a reduction in the proportion of diseased plants in a given region over a given period of time) are not necessarily correlated (Burdon, Zhan, Barrett, Papaïx, & Thrall, 2016; Burdon et al., 2014; Johnson, 1984). Many empirical and modelling studies have demonstrated the epidemiological efficiency of some strategies to control plant disease, especially mixtures (Borlaug, 1953; Calonnec, Goyeau, & de Vallavieille‐Pope, 1996; Garrett & Mundt, 2000; Huang, Sun, Wang, Luo, & Ma, 2012; Jensen, 1952; Mundt, Sackett, & Wallace, 2011; Power, 1991; Zhu et al., 2000). There is also empirical evidence that high fragmentation (Condeso & Meentemeyer, 2007; Fleming, Marsh, & Tuckwell, 1982) or high biodiversity (Haas, Hooten, Rizzo, & Meentemeyer, 2011) at the landscape scale can impede disease spread. Such findings suggest the potential utility of cropping mosaics.

In contrast, realistic assessment of the durability of a given strategy at the landscape scale requires the deployment of major gene resistance across large areas over multiple years, and is consequently much less experimentally tractable. We are aware of only one empirical study designed to compare some of the main categories of deployment (Djian‐Caporalino et al., 2014). This study evaluated the ability of mixtures, rotations and pyramiding of two different resistance sources to control root‐knot nematode of pepper, in both controlled and field conditions. In this context, pyramiding was found to be the best strategy, followed by rotations, and finally mixtures. As a complement to experimentation, modelling is a useful tool to compare the durability and epidemiological efficiency of different strategies and to explore the wide range of spatiotemporal deployment options. To date, no such global comparison, using a single eco‐evolutionary framework and standardized assumptions, exists (REX Consortium 2013, 2016).

The objective of this study is to compare the four main categories of deployment strategies described above for situations where two major resistance genes with a complete efficiency (i.e., they confer immunity) are deployed, and address the following questions:

1. How do evolutionary and epidemiological outcomes vary across different categories of resistance deployment strategies?
2. What are the impacts of landscape organization (proportion of different cultivars planted, and their spatial or temporal aggregation) and pathogen evolutionary ability (mutation probability and associated fitness costs) on the performance of different strategies?
3. Under what conditions it is possible to achieve both evolutionary and epidemiological control of pathogens (i.e., resistance that is both durable and efficient)?

**Figure 1:**
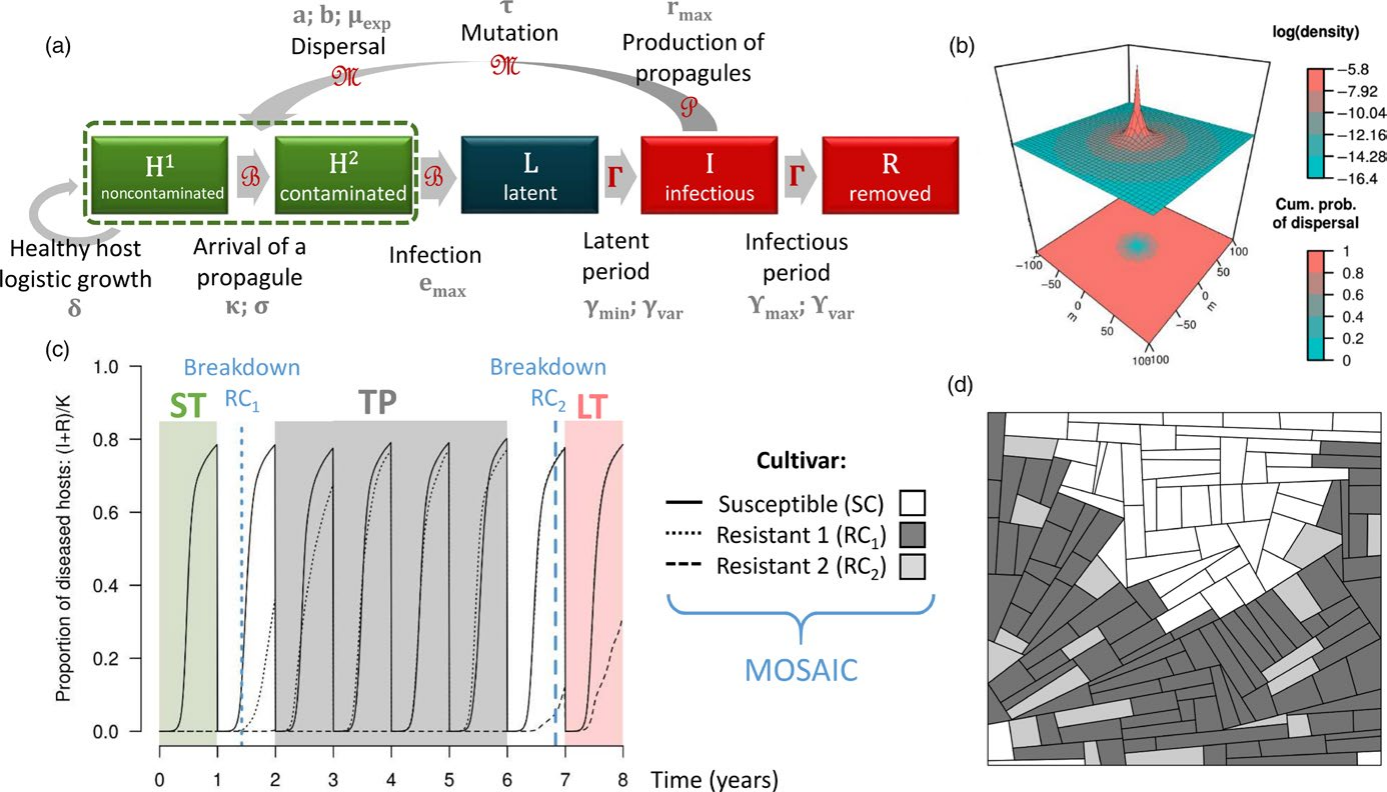
Model overview. (a) Model architecture. To avoid any confusion with the “susceptible” cultivar, the SEIR structure is labelled HLIR for “healthy‐latent‐infectious‐removed.” Healthy hosts can be contaminated by propagules and may become infected. Following a latent period, infectious hosts produce new propagules, which may mutate and disperse across the landscape. At the end of the infectious period, infected hosts become epidemiologically inactive. Qualitative resistance prevents transition to the latent infected state (L). Green boxes indicate healthy hosts, which contribute to crop yield and host growth, in contrast to latent hosts (dark blue box) and diseased hosts (i.e., symptomatic, red boxes). Parameters associated with epidemiological processes are indicated in grey and detailed in Table 1. Distributions used to simulate stochasticity in model transitions are indicated in red; B: binomial, Γ: gamma, P: Poisson, M: multinomial. Host growth is deterministic. (b) Two‐dimensional representation of the power‐law dispersal kernel calibrated for rust pathogens (see equation in Table 1; *μ*_exp_ = 20 m; *a *=* *40; *b *=* *7). Top panel indicates the logarithm of the probability to disperse from the origin to any point of the landscape; bottom panel indicates the cumulative probability of dispersing over a given distance. (c,d) Example of simulation with two major resistance genes deployed as a mosaic: (c) dynamic of diseased hosts and (d) landscape (*φ*_1_ = 2/3; *φ*_2_ = 5/6; *α*_1_ = high; *α*_2_ = low). Blue vertical lines indicate the durability of the two resistant cultivars. These lines delineate the three periods used to compute epidemiological outputs from AUDPC: short‐term (ST, green area), transitory period (TP, grey) and long‐term (LT, red)

To motivate this work, we focus on crop resistance to rust pathogens (fungi of the genus *Puccinia*), although our general conclusions are likely to have broader implications. Many major resistance genes against rust pathogens have been described, but also quickly overcome after deployment in the field (Boyd, 2005; Park, 2008; Thompson & Burdon, 1992). We investigate the questions above using a generic spatially explicit stochastic model, which simulates the spread of epidemics across an agricultural landscape and the evolution of a pathogen in response to the deployment of host resistance (Figure 1). This model, described in a previous study (Rimbaud, Papaïx, Rey, Barrett, & Thrall, 2018) and implemented in the R package *landsepi*, is flexible enough to vary resistance sources, deployment categories and epidemiological, evolutionary and landscape parameters (see Supporting information Videos [Link], [Link], [Link], [Link] for examples). In particular, the model was parameterized to roughly represent rust diseases of cereal crops (Table 1, see also Supporting information Text S1 in Rimbaud, Papaïx, Rey, Barrett et al. (2018)).

**Table 1:** Summary of model parameters and values for rust pathogens

Notation	Parameter	Value
Simulation parameters		
Y	Number of simulated years	48 years^a^
*T*	Number of time‐steps in a cropping season	120 days/year
Initial conditions and seasonality		
${C_{v}}^{0}$	Plantation host density of cultivar v	0.1/m^2^ a
$C_{v}^{\max}$	Maximal host density of cultivar v	2/m^2^ a
δ_v_	Host growth rate of cultivar v	0.1/day^a^
ϕ	Initial probability of infection	5.10^−4^
λ	Off‐season survival probability	10^−4^
Pathogen aggressiveness components		
*e*_max_	Maximal expected infection rate	0.40/spore
γ_min_	Minimal expected latent period duration	10 days
γ_var_	Variance of the latent period duration	9 days
*Υ*_max_	Maximal expected infectious period duration	24 days
*Υ*_var_	Variance of the infectious period duration	105 days
*r*_max_	Maximal expected propagule production rate	3.125 spores/day
Pathogen dispersal		
g(.)	Dispersal kernel	Power‐law function^a^
*a*	Scale parameter	40
*b*	Width of the tail	7
π(.)	Contamination function	Sigmoid curve^a^
κ	Related to position of the inflexion point	5.33
σ	Related to position of the inflexion point	3

*Notes*

See Supporting information Text S1 in (Rimbaud, Papaïx, Rey, Barrett et al., 2018) for calibration details. Epidemic processes associated with some of the parameters are illustrated in Figure 1.

When resistance is deployed within crop rotations, 48 years correspond to 24, 12 or 8 cycles for low, moderate and high value for *α*_2_, respectively.

Same value for all cultivars.

$g\left(\left|\right|z^{\prime }-z\left|\right|\right)=\frac{\left(b-2)(b-1\right)}{2\pi a^{2}}\cdot {\left(1+\frac{\left|\right|z^{\prime }-z\left|\right|}{a}\right)}^{-b}$ with $\left|\right|z^{\prime }-z\left|\right|$ the Euclidian distance between locations *z* and *z*′ in fields *i* and *i*′, respectively; the mean dispersal distance is given by: $\frac{2a}{\left(b-3\right)}$ =20 m, but long‐distance dispersal may also occur.

a $\pi \left(x\right)=\frac{1-e^{-\kappa x^{\sigma }}}{1-e^{-\kappa }}$ with *x* the proportion of healthy hosts in the host population. The position of the inflexion point of this sigmoid curve is given by the relation $x_{o}={\left((\sigma -1)/\kappa \sigma \right)}^{\frac{1}{\sigma }}\approx 0.5$.

## 2 METHODS

### 2.1 Model description

We used a stochastic, spatially explicit demogenetic model fully described in a previous study (Rimbaud, Papaïx, Rey, Barrett et al., 2018). It simulates the clonal reproduction, spread and evolution of a pathogen in an agricultural landscape over multiple cropping seasons. The model is based on a SEIR (“susceptible‐exposed‐infectious‐removed”) structure with a discrete time step. Demographic stochasticity is considered for each transition between compartments using specific probability distributions (Figure 1a): (a) Propagules contaminate healthy hosts depending on their local density and a binomial distribution; (b) contaminated hosts are infected according to an infection rate and a binomial distribution; (c) infected hosts become infectious after a latent period drawn from a gamma distribution; (d) infectious hosts produce propagules according to their reproduction rate and a Poisson distribution; (e) propagules may mutate to acquire infectivity and disperse across the landscape, according to multinomial distributions; and (f) infectious hosts are removed after an infectious period drawn from a gamma distribution. In this model, an “individual host” can be considered as a foliar site where a propagule can land and potentially trigger the development of a localized infection.

In this study, the model is parameterized to approximate biotrophic foliar fungal diseases as typified by rusts of cereal crops, caused by fungi of the genus *Puccinia* (see details on model calibration in Supporting information Text S1 in Rimbaud, Papaïx, Rey, Barrett et al., 2018). Within these pathosystems, spores (i.e., propagules) are produced by sporulating lesions, which develop on the leaves of infected hosts, and are dispersed by wind. The probability of pathogen dispersal from one field to another field of the landscape is computed by integrating a power‐law function (Figure 1b) over all pairs of points belonging to the two considered fields, normalized by the surface of the source field.

It is considered that a cultivar carrying a major resistance gene is immune to disease, unless the pathogen has acquired an infectivity gene via mutation (corresponding to the “gene‐for‐gene” concept and described in many plant–pathogen interactions, especially cereal rusts; Leonard, 1977; Thompson & Burdon, 1992). For infectivity gene g, the mutation probability *τ*_g_ depends on many factors including the number of genetic mutations per generation per base pair (i.e., the classic “mutation rate” of empirical studies), the number and nature of required genetic mutations, and the potential dependency between these mutations. The acquisition of such infectivity leads to breakdown of the associated major resistance gene. However, such evolution may be penalized by a fitness cost on susceptible hosts (Brown, 2015; Laine & Barrès, 2013; Leach, Vera Cruz, Bai, & Leung, 2001; Thrall & Burdon, 2003). Therefore, in our model, pathogens carrying infectivity genes may have reduced infectivity on susceptible hosts relative to pathogens that do not carry these genes (fitness cost denoted by *θ*_g_).

Each cropping season consists of host planting, logistic growth and finally harvest, which imposes a potential bottleneck for the pathogen before the next cropping season. Two stochastic algorithms are used to generate and replicate agricultural landscapes with specific features. Landscape structure is randomly generated using a T‐tessellation algorithm (see Papaïx et al., 2014 for details) to control the number and shape of fields. Landscape composition (i.e., cultivar allocation) is randomly simulated using an algorithm based on latent Gaussian fields (see examples in Figure 1d and Supporting information Figure S1 and Rimbaud, Papaïx, Rey, Barrett et al., 2018 for details). Some fields are cultivated with a susceptible cultivar (SC), which is initially infected by the pathogen. In the other fields (whose proportion and level of spatial aggregation are controlled by parameters *φ*_1_ and *α*_1_, respectively), two major resistance genes are deployed according to one of the following strategies:

- Mosaics: two resistant cultivars (RC_1_ and RC_2_, carrying the first and the second major resistance genes, respectively) are assigned to candidate fields with controlled relative proportion (*φ*_2_) and level of spatial aggregation (*α*_2_) (see Supporting information Video S1 for an example simulation);
- Mixtures: both RC_1_ and RC_2_ are allocated to all candidate fields with a controlled relative proportion (*φ*_2_) (see Supporting information Video S2);
- Rotations: RC_1_ and RC_2_ are alternatively cultivated in candidate fields, depending on the number of cropping seasons over which a given cultivar is grown before being rotated (here, *α*_2_ refers to temporal aggregation) (see Supporting information Video S3);
- Pyramiding: all candidate fields are cultivated with RC_12_, a resistant cultivar carrying both resistance sources (see Supporting information Video S4).

Note, in mixtures, the potential decreased growth due to disease in one of the components is not compensated for by increased growth in other components (i.e., all components are considered independent). This assumption may be simplistic but is more parsimonious than those required to simulate compensation processes for host growth in mixtures, especially if the relative proportions of the different components are unbalanced.

Table 1 summarizes model parameters and their value for rust pathogens.

### 2.2 Simulation plan and model outputs

#### 2.2.1 Simulation plan

The model was used to assess evolutionary and epidemiological outcomes for different deployment categories and a wide range of options to deploy two major resistance genes. In addition to the category of resistance deployment (mosaic, mixture, rotation, pyramiding), we varied the proportion of fields where resistance is deployed (*φ*_1_, five values) and their level of spatial aggregation (*α*_1_, three values). We also varied the relative proportion of RC_2_ (*φ*_2_, five values for mosaics and mixtures) or its level of spatial/temporal aggregation (*α*_2_, three values for mosaics and rotations). To simulate different levels of pathogen evolutionary potential, we varied the mutation probability (*τ*, two values) and associated fitness cost (*θ*, five values) with the same characteristics for both major genes (i.e., *τ*_g_ = *τ* and *θ*_g_ = *θ* ∀ g ϵ{1;2}) and assuming independence between mutations. The values for the mutation probability were selected to simulate two contrasted situations. Trial simulations showed that when *τ* = 10^−7^, a cultivar carrying a single major gene is generally overcome in less than 48 years, but a cultivar carrying a pyramid of two major genes is never overcome. When *τ* = 10^−4^, a cultivar carrying a single major gene is overcome in less than 1 year and a cultivar carrying a pyramid of two major genes is generally overcome in less than 48 years. Thus, these values should ensure breakdown of some resistance sources, while allowing the comparison of different strategies with regard to their respective abilities to mitigate pathogen evolution in the long term.

For each deployment category, the parameters mentioned above were explored using a complete factorial design (Table 2). Simulations were performed using five different landscape structures (about 150 fields, total area: 2 × 2 km^2^, see Supporting information Figure S1 in Rimbaud, Papaïx, Rey, Barrett et al., 2018) and 10 replicates per landscape structure, resulting in 50 stochastic replicates overall, and a total of 180,000 simulations. Every simulation was run for 48 seasons of 120 days each. Trial simulations indicated that this time period was long enough to allow us to differentiate among deployment strategies with regard to their evolutionary and epidemiological performance.

**Table 2:** Simulation plan

Notation	Parameter	Values
Landscape structure		
*J*	Number of fields in the landscape	155; 154; 152; 153; 156^c^
Landscape organization^a^		
*φ*_1_	Cropping ratio of fields where resistance is deployed: $\varphi_{1}=\frac{{\text{RC}}_{1}+{\text{RC}}_{2}}{\text{SC}+{\text{RC}}_{1}+{\text{RC}}_{2}}$	1/6; 2/6; 3/6; 4/6; 5/6
*α*_1_	Level of spatial aggregation of fields where resistance is deployed (RC_1_ and RC_2_)	Low; moderate; high
* φ*_2_	Relative cropping ratio of RC_2_: $\varphi_{2}=\frac{{\text{RC}}_{2}}{{\text{RC}}_{1}+{\text{RC}}_{2}}$	1/6; 2/6; 3/6; 4/6; 5/6^c^
*α*_2_	Relative level of spatial/temporal aggregation of RC_2_	Low; moderate; high^b^
Pathogen evolutionary ability		
*τ*_g_	Mutation probability for infectivity gene g^c^	10^−7^; 10^−4^
*θ*_g_	Fitness cost of infectivity gene g	0.00; 0.25; 0.50; 0.75; 1.00^c^

*Notes*

A susceptible (SC), a resistant cultivar (RC_1_) and possibly a second resistant cultivar (RC_2_) are assigned to fields according to one of the four deployment categories (mosaic, mixture, rotation and pyramids). For each deployment category, parameters related to landscape organization and pathogen evolutionary ability are varied according to a complete factorial design. Every simulation is replicated 10 times × 5 landscape structures to account for stochasticity, resulting in a total of 180,000 simulations.

See Supporting information Figure S1 in Rimbaud, Papaïx, Rey, Barrett et al. (2018) for illustrations of landscape structures generated using a T‐tessellation algorithm, and see Papaïx et al. (2014) for details on the algorithm.

a Crop cultivars are allocated using an algorithm based on latent Gaussian fields to control proportion and level of spatial aggregation of each cultivar; see Supporting information Figure S1 of the present article for illustrations, and see Rimbaud, Papaïx, Rey, Barrett et al. (2018) for details on the algorithm.

For mosaics and mixtures, only.

b For mosaics and rotations, only. In crop rotations, cultivars are rotated every year (*α*_2_ = low), every 2 years (*α*_2_ = moderate) or every 3 years (*α*_2_ = high).

Probability for a propagule to change its infectivity on a resistant cultivar carrying major gene g.

c Same value for all infectivity genes. *θ*_g_ = 0 means absence of cost of infectivity, and *θ*_g_ = 1 means the complete loss of infectivity of adapted pathogens on the susceptible cultivar.

#### 2.2.2 Model outputs

At the end of a simulation run, the results were evaluated using a set of evolutionary and epidemiological outputs (listed in Table 3 and detailed in Rimbaud, Papaïx, Rey, Barrett et al., 2018). Evolutionary outputs characterize three steps required to overcome major gene resistance: (a) first appearance of mutants, (b) initial migration to resistant hosts and infection and (c) broader establishment in the resistant host population (i.e., the first time when the number of infections of resistant hosts exceeds a threshold above which extinction in a steady environment becomes unlikely). Epidemiological outputs were evaluated using the area under the disease progress curve (AUDPC) to measure disease severity (on a specific cultivar or on the whole landscape) across the whole simulation run or across characteristic periods of pathogen adaptation to resistance: (a) the initial short‐term period when all major resistance genes were still effective; (b) when appropriate, a transitory period during which one major gene has been overcome but not the second one (i.e., the deployment strategy was only partially effective); and (c) a longer‐term period when all major resistance genes have been overcome. Figure 1c provides an example of a simulation run and delimitation of these periods.

**Table 3:** List of model outputs computed at the end of a simulation run

Notation	Output
Evolutionary outputs (related to resistance durability)^a^^,^ b	
Mut_1_	First appearance of a mutant carrying infectivity gene 1
Mut_2_	First appearance of a mutant carrying infectivity gene 2
Mut_12_	First appearance of the superpathogen^c^
Inf_1_	First infection of a resistant host by a mutant carrying infectivity gene 1
Inf_2_	First infection of a resistant host by a mutant carrying infectivity gene 2
Inf_12_	First infection of a resistant host by the superpathogen^c^
Dur_1_	Broader establishment of a mutant carrying infectivity gene 1 in the resistant host population
Dur_2_	Broader establishment of a mutant carrying infectivity gene 2 in the resistant host population
Dur_12_	Broader establishment of the superpathogen^c^ in the resistant host population
Epidemiological outputs computed from AUDPC (related to epidemiological efficiency)^d^	
AUDPC_SC_	Disease severity on the susceptible cultivar
AUDPC_RC1_	Disease severity on resistant cultivar 1, carrying major resistance gene 1
AUDPC_RC2_	Disease severity on resistant cultivar 2, carrying major resistance gene 2
AUDPC_ST_	Short‐term control, computed on the susceptible cultivar from the beginning of the simulation until one of the major resistance gene is overcome^e^
AUDPC_TP_	Control during the transitory period when only one major resistance gene is overcome, computed on the susceptible cultivar^e^
AUDPC_LT_	Long‐term control, computed on the whole landscape from the time both major resistance genes are overcome until the end of the simulation run^f^
AUDPC_tot_	Global control, computed on the whole landscape across the whole simulation run

*Notes*

a When a duration exceeds the simulation run (48 years, i.e., 5,760 time‐steps), it is set at 48 years + 1 day.

b In the pyramiding strategy, the resistant cultivar carries both major resistant genes 1 and 2, thus Inf_1_ = Inf_2_ = Inf_12_ and dur_1_ = dur_2_ = dur_12_.

c The superpathogen carries both infectivity genes 1 and 2 and is able to overcome both major resistance genes 1 and 2.

d In the pyramiding strategy, AUDPC_RC1_ = AUDPC_RC2_ and AUDPC_TP_ cannot be computed.

Cannot be computed if a major gene is overcome before the end of the first cropping season.

e Cannot be computed if the second major gene is overcome less than 2 years after the first major gene.

f Cannot be computed if all major genes have not been overcome by the end of the simulation.

### 2.3 Statistical analyses

#### 2.3.1 Polynomial regressions

For every deployment category and mutation probability, the number of time‐steps until mutants carrying the first, the second or both infectivity genes became established in resistant host population were fitted by generalized linear models. We used a Poisson regression with logarithm as the link function, and third‐degree Legendre polynomials including interactions up to second order, and restricted to polynomial terms of up to degree 3. The explaining variables were the cropping ratio (*φ*_1_), the level of spatial aggregation (*α*_1_), the cost of infectivity (*θ*), and, when appropriate, the relative cropping ratio (*φ*_2_) and the relative level of aggregation (*α*_2_). In these analyses, *α*_1_ and *α*_2_ were considered as quantitative variables (low = 1, intermediate = 2, high = 3), and all explaining variables were rescaled between −1 and 1 (definition domain of Legendre polynomials). In the same way, AUDPC values corresponding to epidemiological outputs (when available, short‐term control, control during the transitory period, long‐term and overall control) were normalized by the average AUDPC obtained in a fully susceptible landscape (AUDPC_0_ = 0.38) and fitted by linear models with Legendre polynomial regressions. Because these polynomials are orthogonal, they could be used to compute sensitivity indices of model parameters and their interactions (polynomial chaos expansion, Sudret, 2008). The main (or “first‐order”) sensitivity index of an input parameter measures its main relative contribution to the variance of the output variable, whereas the total sensitivity index includes its interactions with other parameters. Furthermore, the polynomial regressions were also used to predict each model output from different values of model parameters. Supporting information Table S1 gives metrics of goodness of fit for every regression.

#### 2.3.2 Principal component analyses

A principal component analysis (PCA) was performed on model outputs showing nonmissing values. Because some epidemiological metrics could not be computed in many simulations (AUDPC_ST_, AUDPC_TP_ and AUDPC_LT_), global disease severity for the different cultivars (AUDPC_SC_, AUDPC_RC1_ and AUDPC_RC2_) across the whole simulation period was used instead.

The model is written using the C and R languages and is available in the R package *landsepi* (Rimbaud, Papaïx, & Rey, 2018). Within the R (v3.4.0, R Core Team 2012) software, package *ade4* (v1.7‐6, Dray & Dufour, 2007) was used to compute the PCA, and package *orthopolynom* (v1.0‐5, Novomestky, 2013) to compute the Legendre polynomials. One simulation run takes approximately 60 s on a standard desktop computer (Intel® Core™ i5‐5300U).

## 3 RESULTS

Using a factorial design (see Table 2 and Methods for details), we varied model parameters associated with the deployment category (mosaic, mixture, rotation or pyramiding), pathogen evolutionary potential (mutation probabilities and associated fitness costs) and landscape organization (proportion of resistant fields, relative proportion of each major gene present and levels of aggregation and relative aggregation; see Supporting information Figure S1 for examples of simulated landscapes). Here, the durability of major resistance genes was measured by the time to establishment of adapted (mutant) pathogens in the resistant host population (i.e., the point at which extinction in a steady environment becomes unlikely) and also referred to as “time to breakdown.” Epidemiological outcomes were evaluated using the area under the disease progress curve (AUDPC) to measure disease severity across characteristic periods of pathogen adaptation to resistance (see Table 3 for details). Previous work showed that landscape structure (spatial structure of local field boundaries, not to be confused with landscape organization) had no effect on model outputs (see Supporting information Figure S2). Thus, each of the 3,600 different combinations of input parameters was replicated 10 times × 5 landscape structures to account for model stochasticity, resulting in a total of 180,000 simulations.

In a typical manner, a simulation was initiated with the allocation of a susceptible cultivar and two cultivars carrying two major resistance genes (or one cultivar carrying both genes for the pyramiding strategy). Figure 1d provides an example of landscape organization in a mosaic strategy. For this study, we assumed that the initial pathogen population was only adapted to susceptible hosts and that the major resistance genes conferred complete immunity to resistant hosts. However, through mutation, the pathogen can acquire infectivity genes able to overcome the associated major resistance genes. In this work, “infectivity” is defined as in previous studies (Burdon et al., 2014, 2016; Susi, Thrall, Barrett, & Burdon, 2017) as the qualitative ability to infect a resistant host (i.e., it is synonymous with the term ‘virulence’ in plant pathology; however, we prefer to use infectivity, as virulence has different meanings in the plant pathology, parasitology and evolutionary biology literature). Epidemics were simulated using a demogenetic model with SEIR structure (Figure 1a and Methods). The dispersal of mutant pathogens to fields planted with resistant cultivars (see the power‐law dispersal kernel in Figure 1b) may allow infection of resistant hosts and subsequent establishment of infective pathogens in the resistant host population (see an example of disease dynamics in Figure 1c). After 48 years (cropping cycles) of simulation, evolutionary and epidemiological outcomes were characterized by a set of model outputs (see the complete list of output variables in Table 3).

Of the 180,000 simulations, 109 resulted in pathogen extinction before the end of the simulation. In the other simulations, mutant pathogens appeared on average after 0.27 year (min–max: 0.01–1.60) and infected resistant hosts after an average of 5.55 years (0.01–48.00). The mean durability of resistance genes was approximately 12.55 years (0.50–48.00). The epidemiological outputs, all based on the computation of the AUDPC, varied from 0% (i.e., no disease) to 97% (i.e., severe epidemics) of the maximum obtainable in the absence of resistant hosts. Below, we focus on specific factors that drive variability in performance among different resistance deployment strategies.

### 3.1 Evolutionary outcomes

Every simulation resulted in one of four evolutionary outcomes, depending on whether (a) major gene breakdown did not occur, (b) only one gene was overcome, (c) both major genes were overcome by different pathotypes or (d) the two major genes suffered breakdown and, in addition, a superpathogen (able to overcome both major resistance genes) emerged and established in the resistant host population.

#### 3.1.1 Durability of major resistance genes

**Figure 2:**
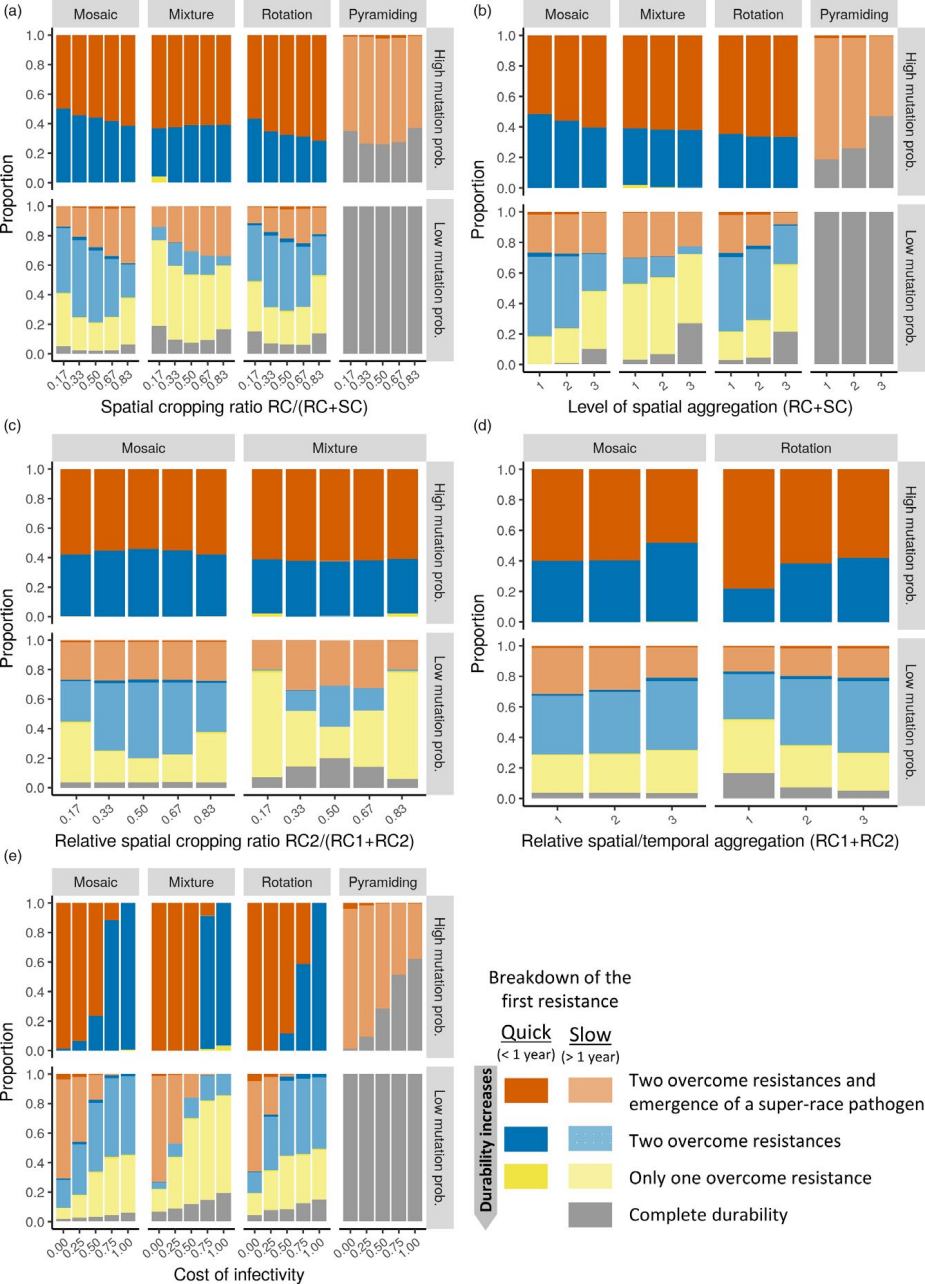
Evolutionary outcomes. Proportion of simulations associated with each of the possible evolutionary outcomes, at high (*τ* = 10^−4^) and low (*τ* = 10^−7^) mutation probabilities. Panels show the effect of the proportion of fields where resistance is deployed (a), their level of spatial aggregation (b), the relative proportion of the second major gene (c), its relative level of spatial (for mosaics) or temporal (for rotations) aggregation (d) and the fitness cost associated with pathogen infectivity (e). SC, susceptible cultivar; RC, resistant cultivars, including the first (RC_1_) and the second (RC_2_) resistance gene. Darker shaded colours refer to situations where resistance breakdown was rapid (<1 year), while faded colours refer to those where resistance breakdown was slower (>1 year)

At high mutation probabilities (*τ* = 10^−4^), almost 100% of the simulations associated with mosaics, mixtures and rotations resulted in the breakdown of both major genes in less than one cropping season, possibly along with the establishment of a superpathogen (Figure 2). In contrast, complete durability was maintained in 30% of the simulations performed with a pyramiding strategy, and in most of the remaining simulations, more than one cropping season was necessary to overcome the pyramid. At low mutation probabilities (*τ* = 10^−7^), the pyramiding strategy was always completely durable, whereas mosaics, mixtures and rotations displayed all possible evolutionary outcomes. The two major genes were more often completely durable under both low and high cropping ratios (proportion of fields cultivated with resistant hosts, Figure 2a), with increasing levels of spatial aggregation of the resistant fields (Figure 2b) and with increasing fitness costs associated with pathogen mutation towards greater infectivity (Figure 2e). In mosaics and especially mixtures, one of the major genes more often remained effective when its relative proportion in resistant fields (relative cropping ratio within resistant fields) was low, to the detriment of the other major gene (Figure 2c). Varying the relative spatial aggregation of the major genes among resistant fields in mosaics did not impact the proportion of simulations where they were overcome, whereas increasing the temporal aggregation in rotations (i.e., time until a cultivar is rotated) led to smaller proportion of simulations where both genes remained completely durable (Figure 2d).

This qualitative assessment of major resistance gene durability was complemented by a quantitative analysis using polynomial regression (see Supporting information Table S1 for goodness‐of‐fit metrics). This analysis showed that the durability of a given gene was mostly influenced by its relative proportion in mosaics and mixtures, by the proportion of resistant fields in the landscape and their level of spatial aggregation in rotations and by these two last parameters as well as the cost of infectivity in pyramids (see the sensitivity analyses in Supporting information Figures S3 and S4).

**Figure 3:**
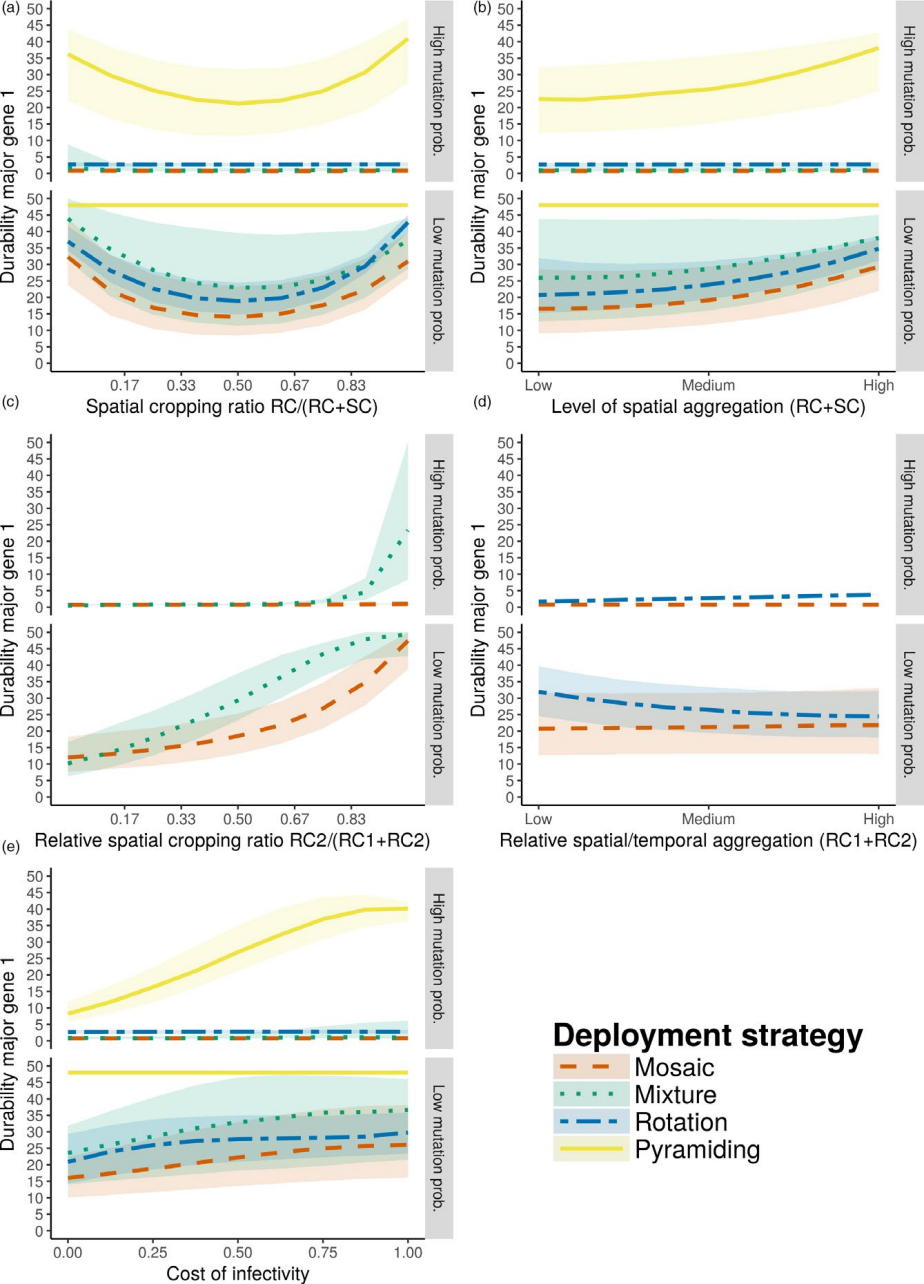
Resistance gene durability. Durability (in years) of the first major resistance gene (Dur_1_) at high (*τ* = 10^−4^) and low (*τ* = 10^−7^) mutation probabilities. Panels show the effect of the proportion of fields where resistance is deployed (a), their level of spatial aggregation (b), the relative proportion of the second major gene (c), its relative level of spatial (for mosaics) or temporal (for rotations) aggregation (d) and the fitness cost associated with pathogen infectivity (e). Curves represent median predictions using third‐degree Legendre polynomials including interactions up to second order within a Poisson generalized linear model; shaded envelopes are delimited by the first and third quartiles. SC, susceptible cultivar; RC, resistant cultivars, including the first (RC_1_) and the second (RC_2_) resistance gene. The second major resistance gene is associated with similar results (see Supporting information Figure S5). Note that when a major resistance gene remains effective during the whole simulation run, its durability is set at 48 years, and also that in pyramids Dur_1_ = Dur_2_ = Dur_12_

Model predictions of the durability of the two major genes corroborated the qualitative analysis with regard to the U‐shaped effect of cropping ratio (Figure 3a and Supporting information Figure S5A), the positive effect of the level of spatial aggregation of resistant fields and the cost of infectivity (Figure 3b,e and Supporting information Figure S5B,E), the small effect of the relative aggregation (Figure 3d and Supporting information Figure S5D), as well as the increased durability of the minority component in mosaic and mixture strategies (Figure 3c and Supporting information Figure S5C). Overall, our results found that pyramiding the two major resistance genes offered the best durability, followed by mixtures, and finally mosaics and rotations. It should be noted that rotations appeared to perform better than mosaics with respect to the durability of the first major resistance gene (Figure 3) but not with regard to the second gene (see Supporting information Figure S5), although both genes were assumed identical. This was actually an artefact of the model, owing to the initial conditions: Rotations were simulated by starting with the second resistant cultivar (RC_2_), so the first resistant cultivar (RC_1_, carrying the first major resistance gene) was not deployed before Year 2, 3 or 4 (depending on the level of temporal aggregation). Thus, the durability of the first major gene (computed from the beginning of the simulation) was slightly overestimated in rotations.

#### 3.1.2 Time to emergence of a superpathogen

At high mutation probabilities, the cost of infectivity had by far the greatest impact on the time to establishment of a superpathogen in the resistant host population (see sensitivity analysis in Supporting information Figure S6A). In the absence of infectivity costs, a superpathogen emerged in almost all simulations. In contrast, when costs were high (i.e., infection rates on susceptible hosts were reduced by 75%), superpathogens emerged in only 11%, 9%, 41% and 49% of the simulations performed with mosaics, mixtures, rotations and pyramids, respectively (Figure 2e). It is interesting to note that, at this level of infectivity cost, mosaics, mixtures and rotations impeded superpathogen establishment more efficiently than pyramids (see Supporting information Figure S6B for model predictions using polynomial regression). At low mutation probabilities, the superpathogen never emerged in pyramids, as previously noted. In the other deployment categories, the superpathogen became established more often with increasing cropping ratios in mosaics and mixtures (Figure 2a), decreasing level of spatial aggregation between susceptible and resistant hosts in rotations (Figure 2b) and especially decreasing costs of infectivity (Figure 2e). The time to establishment was mainly driven by the interaction between the cost of infectivity and other parameters: cropping ratio for all three strategies, relative cropping ratio for mixtures and level of spatial aggregation for rotations (see Supporting information Figure S6A). Increasing costs of infectivity mitigated (towards completely annulling) the effect of the other parameters (see model predictions in Supporting information Figure S6C–G).

### 3.2 Epidemiological outcomes

In a fully susceptible landscape, the average disease severity (represented by the area under disease progress curve, AUDPC_0_) was formerly estimated at 0.38 (Rimbaud, Papaïx, Rey, Barrett et al., 2018), meaning that diseased host (states I and R in Figure 1a) represented an average proportion of 38% of the carrying capacity. In the current study, all computations of AUDPC (see list of model outputs in Table 3 and details in Rimbaud, Papaïx, Rey, Barrett et al., 2018) were expressed relative to AUDPC_0_; hence, they might vary from 0% (i.e., no disease) to 100% (i.e., same disease severity as in a fully susceptible landscape).

#### 3.2.1 Short‐term disease control and control during the transitory period

**Figure 4:**
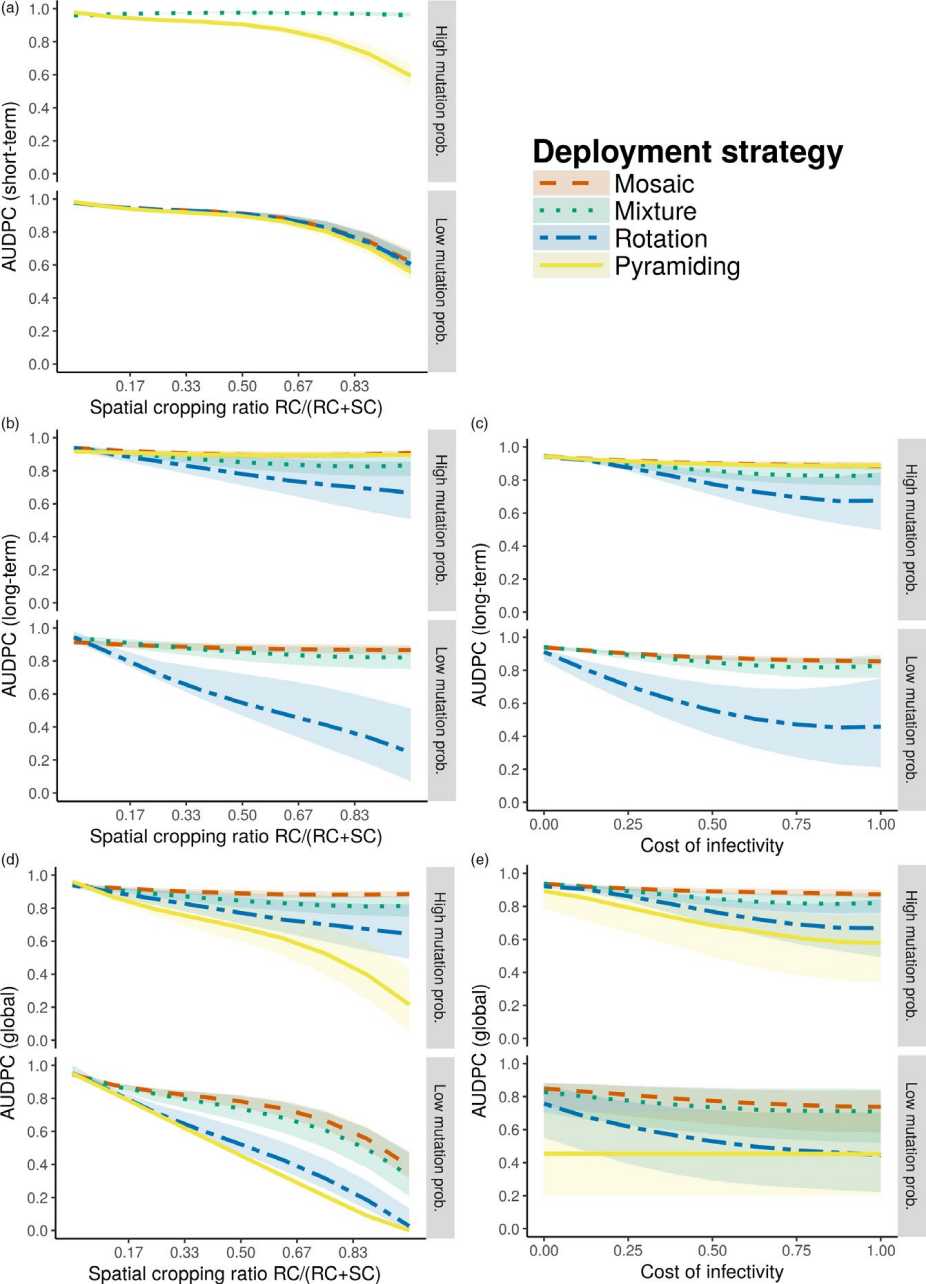
Epidemiological outcomes. Predictions from polynomial regressions, using third‐degree Legendre polynomials including interactions up to second order, of the effect of the proportion of fields where resistance is deployed (a,b,d) or the fitness cost associated with pathogen infectivity (c,e) on different epidemiological outputs at high (*τ* = 10^−4^) or low (*τ* = 10^−7^) mutation probability: AUDPC on the susceptible cultivar in the short‐term period when resistant cultivars are still immune to disease (AUDPC_ST_, a); and AUDPC on the whole landscape computed in the long‐term period when all resistances have been overcome (AUDPC_LT_, b,c) or in the whole simulation (AUDPC_TOT_, d,e). Curves represent the median and envelopes are delimited by the first and third quartiles. SC, susceptible cultivar; RC, resistant cultivars, including the first (RC_1_) and the second (RC_2_) resistance gene. Note in (a) that at high mutation probabilities, mosaics, mixtures and rotations were almost always overcome in less than 1 year; thus, AUDPC_ST_ could not be properly computed

Short‐term control was defined here as the epidemiological protection provided to susceptible hosts by resistant cultivars when the deployment strategy was completely effective and denoted by AUDPC_ST_ (green area in Figure 1c, computed in simulations represented in faded colours in Figure 2, i.e., where resistance durability was greater than 1 year). The epidemiological protection provided by a partially effective deployment strategy (i.e., when only one major resistance gene was overcome) was also computed and denoted by AUDPC_TP_ (grey area in Figure 1c). At high mutation probabilities, these criteria could not be assessed for strategies other than pyramiding, as in these scenarios both major genes were overcome during the first year of simulation in almost all simulations. For low mutation probabilities, sensitivity analyses based on polynomial regressions indicate that only the proportion of fields where resistance was deployed (cropping ratio) had an impact on short‐term control (see Supporting information Figure S7). For the transitory period, the results were very similar, except that the cost of infectivity had a slightly greater influence, with stronger costs amplifying the effect of the cropping ratio (see Supporting information Figure S8). The polynomial regressions show that the different deployment categories performed equally well in the short‐term and the transitory periods, with better control for higher cropping ratios (Figure 4a and Supporting information Figure S8B).

#### 3.2.2 Long‐term disease control

The long‐term control, denoted by AUDPC_LT_, characterized disease severity of the whole landscape once all resistances had been overcome (red area in Figure 1c, computed in simulations represented in blue and orange in Figure 2, i.e., where all resistances have been overcome). For both high and low mutation probabilities, the sensitivity analyses highlight the key role of cropping ratio, the cost of infectivity and their interaction (see Supporting information Figure S9). As shown by the polynomial regressions, the higher the cropping ratio and the cost of infectivity, the better the epidemiological control in the long term. In contrast to previous metrics of epidemiological control, the rotation of two major genes performed significantly better than the other strategies in this context (Figure 4b,c).

#### 3.2.3 Global control

The overall efficiency of a deployment strategy was assessed using the AUDPC of the whole landscape, averaged across the entire simulation run (AUDPC_TOT_). The sensitivity analyses highlight the same key parameters as for long‐term control, except that the cost of infectivity was less influential when the mutation probability was low (see Supporting information Figure S10). Better global epidemiological control was obtained with higher cropping ratios, and, to a lesser extent, with higher costs of infectivity (excepting pyramiding at low mutation probabilities). Globally, the polynomial regressions indicate that pyramids of two major genes resulted in better overall control than rotations, followed by mixtures and mosaics (Figure 4d,e).

### 3.3 Trade‐offs between evolutionary and epidemiological disease control

Principal component analysis (PCA) was performed on the simulation results to investigate the relationships between the various model outputs. It should be noted, however, that only model outputs showing nonmissing values could be included in this analysis; hence, global disease severity for the susceptible (AUDPC_SC_) and resistant cultivars (AUDPC_RC1_ and AUDPC_RC2_) across the whole simulation period was used instead of short‐term control (AUDPC_ST_), control during the transitory period (AUDPC_TP_) or long‐term control (AUDPC_LT_) of the disease.

#### 3.3.1 Evolutionary and epidemiological axes

The projection of model outputs on the two main axes explained 64% of the total variance (see Supporting information Figure S11, inset). Factors mainly contributing to the horizontal axis included time to first appearance, first infection and broader establishment of mutants carrying the first, the second or both (i.e., the superpathogen) infectivity genes in the resistant host population (see Supporting information Figure S11). Disease severity on the two resistant cultivars also contributed to this axis and was negatively correlated with the latter outputs related to resistance durability (i.e., the time period during which the resistant cultivars were immune to disease). In contrast, the vertical axis was mainly determined by disease severity on the susceptible cultivar. These results suggest that outputs related to resistance durability and epidemiological protection of the susceptible cultivar were not necessarily correlated. Thus, the two main axes can be referred to as the evolutionary and the epidemiological axes. Global control (AUDPC_Tot_) contributed to both axes.

#### 3.3.2 Effect of different deployment strategies

**Figure 5:**
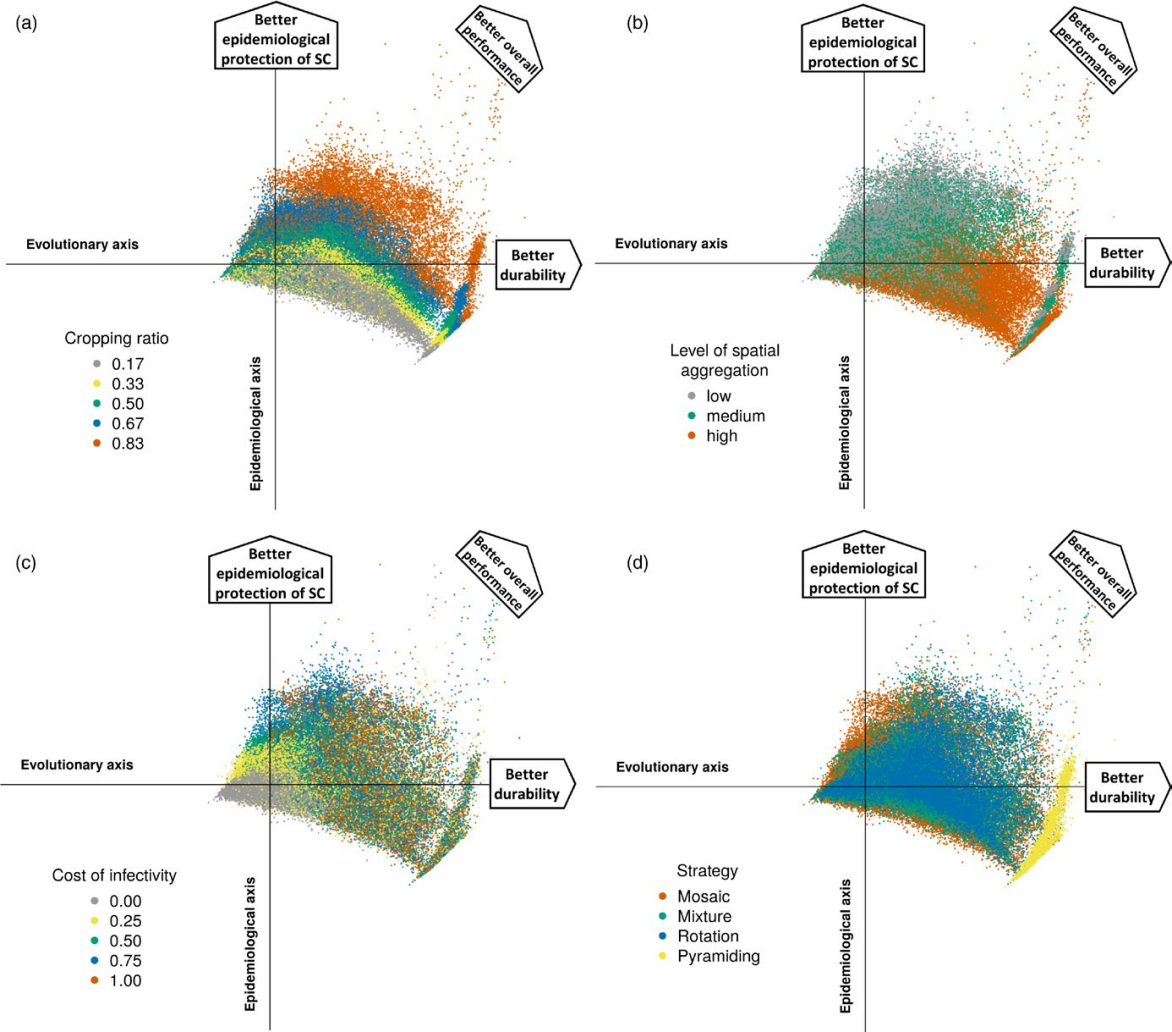
Principal component analysis of model outputs. Projection of the simulation results on the two main axes (total explained variance: 64%), with colour codes reflecting: (a) the proportion of fields where resistance was deployed; (b) their level of spatial aggregation; (c) the fitness cost associated with pathogen infectivity; and (d) the category of the deployment strategy. For legibility, only dots associated with low mutation probabilities (*τ* = 10^−7^) are represented (see Supporting information Figure S12 for dots associated with high mutation probabilities)

Pyramiding offered the best durability at low mutation probabilities, but other deployment strategies provided better epidemiological protection of the susceptible cultivar across the whole simulated period (Figure 5d and Supporting information Figure S12D). Both resistance durability and epidemiological protection of the susceptible cultivar were improved with a greater proportion of resistant fields (Figure 5a and Supporting information Figure S12A) and, to a lesser extent, stronger costs of infectivity (mostly at high mutation probabilities, Figure 5c and Supporting information Figure S12C). Finally the effect of spatial aggregation of resistant fields showed a larger contrast between evolutionary and epidemiological outcomes: Higher degree of aggregation led to better durability, but weaker disease control on the susceptible cultivar (Figure 5b and Supporting information Figure S12B).

## 4 DISCUSSION

Here, we investigated a suite of strategies that have the potential to constrain the evolutionary potential of pathogens to overcome plant disease resistance. It is interesting that some of these strategies have counterparts in the application of pesticides or drugs treatments (van den Bosch & Gilligan, 2008; Gilligan, 2008). Indeed, mosaics of different cultivars are equivalent to treatments of different fields or animals (including humans), crop rotations refer to the periodic application of molecules and pyramiding matches with the combination of molecules in a single treatment. Previous empirical and modelling studies have variously evaluated the performance of all these strategies in controlling pathogens, but few of these allowed direct comparisons between all possible categories of strategies (REX Consortium 2013, 2016). Recently, a global approach has been proposed to compare periodic applications, treatment of different patients and combination strategies for antibiotic treatment in hospitals (Tepekule, Uecker, Derungs, Frenoy, & Bonhoeffer, 2017). In this approach, the combination therapy outperformed the other strategies in most cases. Nevertheless, in the context of plant disease, different results may be expected owing to the spatial structuration of plant epidemics. In the plant pathology literature, some studies compared two categories of resistance deployment strategies (Djidjou‐Demasse, Moury, & Fabre, 2017; Kiyosawa, 1972; Koller et al., 2018; Sapoukhina, Durel, & Le Cam, 2009; Skelsey, Rossing, Kessel, & van der Werf, 2010), and very few have compared three categories of strategies (Djian‐Caporalino et al., 2014; Lof, de Vallavieille‐Pope, & van der Werf, 2017). Moreover, the durability of resistance and the epidemiological control it provides have rarely been considered jointly, although these are not necessarily correlated (van den Bosch & Gilligan, 2003; Burdon et al., 2014; Fabre, Rousseau, Mailleret, & Moury, 2015; Papaïx et al., 2018). Here, we used a previously developed spatiotemporal simulation model (Rimbaud, Papaïx, Rey, Barrett et al., 2018), to examine both evolutionary and epidemiological outcomes for four major categories of deployment strategies: mosaics, mixtures, rotations and pyramids. For each strategy, a range of deployment options was explored with regard to the proportion and level of aggregation of the different cultivars. For the pathogen, mutation probability and the cost of infectivity were also varied to provide some consideration of these important biological features. It is important to note that we arbitrarily selected two values for the mutation probability to investigate the deployment strategies in two contrasted situations and that our intent was to compare different deployment strategies rather than provide an absolute prediction of the durability and efficiency of a particular strategy.

The model was parameterized to broadly represent rust diseases of cereal crops with a focus on strategies involving the deployment of two major resistance genes in areas where the pathogen was already present (although not initially adapted to host resistance). We recognize that our focus on major gene resistance conferring immunity to infection by nonadapted pathogens means that we are assessing only a subset of the types of major resistance genes effective against stem, leaf and stripe rust of cereal crops (McIntosh, Wellings, & Park, 1995). Other major resistance genes (either “weak” major genes coding for NLR proteins or genes involved in adult plant resistance) may provide incomplete protection allowing some pathogen reproduction (Burdon et al., 2014). The possible consequences of the simultaneous use of major resistance genes with different expression profiles (with the potential for contrasting and fluctuating selection on the pathogen) will be the focus of a subsequent study.

### 4.1 No deployment strategy is universally optimal

#### 4.1.1 High durability of pyramids

Our results are consistent with previous empirical and modelling studies suggesting that, in absence of preadapted pathogens, pyramids of resistance genes (or, similarly, combination of molecules in the context of pesticide applications) outcompete other deployment strategies with regard to durability (Djian‐Caporalino et al., 2014; REX Consortium 2013). In real‐world pathosystems, pyramids of resistance genes are expected to show good durability because of the low probability that the pathogen will simultaneously acquire all of the mutations required to overcome multiple major genes and the potential accumulation of fitness costs associated with these mutations (Leach et al., 2001). Both factors contributed to the durability of our simulated pyramids. At low mutation probabilities, mutants with single infectivity appeared within 1 year, whereas mutants with double infectivities (i.e., superpathogens) never appeared (see Supporting information Figure S13A,C). At high mutation probabilities, superpathogens appeared quickly (on average after 0.5 year), but establishment within the population took much longer. In particular, superpathogens took an average of 14 years to be transmitted to resistant hosts and did not become established on average before 24 years (Supporting information Figure S13B and Figure 3). The delays between appearance, infection of resistant hosts and subsequent establishment are because mutant pathogens must survive the end of season bottleneck and also because they first appear in susceptible fields, where they may suffer a cost of infectivity compared to noninfective pathogens, before dispersal to resistant fields.

It is important to note that the scenario we simulated favoured pyramiding durability (Stam & McDonald, 2018). We assumed no prior adaptation of the pathogen to the deployed resistances, so infective pathogens could only appear through mutation. In the real world, complex pyramids are often developed via the incremental addition of major genes to ones that have already been deployed elsewhere (Burdon et al., 2016). In this context, infectivity towards some of the major genes in the pyramid may already be present in a pathogen population. As shown in recent modelling studies, the initial presence of preadapted pathogens can have a dramatic impact on the durability of the pyramid compared to other strategies (Djidjou‐Demasse et al., 2017; Lof et al., 2017). Moreover, mutations towards multi‐infectivity were considered independent, and our model does not currently include pathogen sexual reproduction. Synergistic mutations and sexual reproduction may facilitate acquisition and reassortment of infectivity genes in pathogen populations and further accelerate the breakdown of pyramids (McDonald & Linde, 2002). Sexual reproduction is uncommon in cereal rust pathogens at least in some parts of the world (Park, 2008), but it should be accounted for where there is a real possibility that it contributes to diversity (Ali et al., 2014; Groth & Roelfs, 1982).

#### 4.1.2 Mosaics, mixtures and rotations can mitigate superpathogen emergence

Pathogen adaptation to a pyramid results in the breakdown of all of the component resistance genes. In contrast, when resistance sources are deployed in different cultivars, there are intermediate evolutionary outcomes between the complete durability of all cultivars and establishment of a superpathogen able to infect all hosts. Our results indicate that, at high mutation probabilities, when the cost of infectivity is also high, rotations, and particularly mosaics and mixtures, were better able to prevent or at least delay the establishment of a superpathogen than pyramids (Figure 2e and Supporting information Figure S6B). This can be explained by the fact that the superpathogen accumulates fitness costs (due to the accumulation of mutations). The higher these costs, the less the superpathogen is adapted to cultivars carrying single resistance genes, and thus the more it relies on the presence of the cultivar carrying multigene resistance (absent in the mosaics, mixtures and rotations we simulated). This disruptive selection, based on host genetic diversity, exploits these fitness differences to favour local host specialization of the pathogen and constrain the emergence of generalists (Barrett, Kniskern, Bodenhausen, Zhang, & Bergelson, 2009). For example, in China, a traditional century‐old rice agrosystem, based on mosaics of rice cultivars carrying various resistance sources and cultivated using appropriate cropping ratios, induced a high level of specialization of *Magnaporthe oryzae* on locally grown rice cultivars (Liao et al., 2016). This specialization, due to the fitness costs associated with local adaptation of the pathogen, is likely the main contributor to the successful control of rice blast in this agrosystem.

#### 4.1.3 All strategies offer the same short‐ and mid‐term epidemiological protection

When all resistances were still effective, all resistant cultivars were considered immune to the disease. In this context, it was not surprising to observe similar short‐term epidemiological outcomes from different deployment strategies, all of them being equivalent to a mosaic of a susceptible and a resistant cultivar (Figure 4a). Therefore, short‐term epidemiological control depended more on the proportion of fields where resistance was deployed (see also below). We obtained similar results with partially effective strategies (i.e., only one major gene was overcome, Supporting information Figure S8B). All these results show that for a given organization of an agricultural landscape (i.e., particular cropping ratio and level of aggregation), disease dynamics on the susceptible cultivar (as represented by averaged AUDPC values) were largely unaffected by the way the major genes were deployed in the other fields.

#### 4.1.4 Rotations decrease losses once all resistances have been overcome

In a recent article, Djidjou‐Demasse et al. (2017) compared mosaics and pyramiding strategies in a scenario where all pathotypes (including infective ones) were initially present in pathogen population (although not with the same frequency). They found that mosaics were at least as good as pyramids with regard to an AUDPC‐based criterion which may, to some extent, be compared to our long‐term epidemiological control, once all major resistance genes had been overcome (AUDPC_LT_). With respect to this criterion, our mosaics and pyramids of two major resistance genes performed similarly, and mixtures were slightly better (Figure 4b,c). These differences may be attributed to the fact that in the first study (Djidjou‐Demasse et al., 2017), mosaics outperformed pyramids mostly when three or more major resistance genes were deployed, and when there was high interfield pathogen transmission. It could be interesting to assess the impact of the dispersal kernel (parameterized here to rust diseases, although with some uncertainty on the likelihood of long‐dispersal events, see Supporting information Text S1 in Rimbaud, Papaïx, Rey, Barrett et al., 2018) on our findings. Our results also show that rotations performed significantly better than the other strategies. Once all resistances are overcome, the system becomes equivalent to a set of genetically diverse susceptible cultivars and diverse pathogen populations. However, with crop rotations, a well‐adapted specialist pathogen can lose its associated host at the end of a cropping season. This pathogen then becomes maladapted to its new environment, which imposes severe bottlenecks and increases the likelihood of extinction events.

To disentangle the effects of spatial and temporal diversity, in our simulations the two resistant cultivars were never present at the same time in rotations (one replaced the other). Real agricultural landscapes are more complex, where neighbouring fields are sown with rotating cultivars in such a way that the whole system consists of a temporally dynamic mosaic which essentially combines our definitions of mosaics and rotations. In such systems, all cultivars may be present simultaneously in the landscape (although their locations may change from year to year). In this situation, in contrast to our simulation framework, even if rotations remove a host in space, specialist pathogens may disperse to fields where the cultivar is newly grown. The extent to which this reduces the performance of rotations (as compared to our results) would at least partly depend on pathogen dispersal and survival abilities, two key life history features (Barrett, Thrall, Burdon, & Linde, 2008; Buoro & Carlson, 2014). This further suggests that the efficacy of rotations may well vary for different kinds of pathogens.

#### 4.1.5 Pyramids and rotations had the best global efficiencies

This result can be explained by the fact that the global control provided by each category of resistance deployment was computed for the entire landscape over the whole simulation run. As all strategies had the same epidemiological performance during the short‐term and the transitory periods, global control was mostly correlated with the durability of resistance (during which resistant cultivars did not contribute to the global AUDPC) and long‐term epidemiological control. Therefore, promising deployment strategies would consist of rotating different pyramids of resistance genes, provided these genes have not been already overcome somewhere.

### 4.2 Landscape organization impacts both durability and epidemiological efficiency

#### 4.2.1 Impact of cropping ratio and spatial aggregation

This study emphasizes the impact of landscape organization on the epidemiological and evolutionary performance of different resistance deployment strategies. In mosaics, high proportions of fields cultivated with a resistant cultivar (Fabre et al., 2015; Papaïx et al., 2014, 2018) or a nonhost species (Skelsey et al., 2010) with weak levels of aggregation (or strong connectivity between susceptible and resistant fields) have been shown to favour good epidemiological control. The same conclusions emerged for mixtures (Suzuki & Sasaki, 2011; Xu & Ridout, 2000). The present study is consistent with these conclusions and extends them to rotation and pyramiding strategies (Figures 4 and 5c,d). When the proportion of resistant fields increases, the proportion of hosts suitable for pathogen infection decreases and disease spread is reduced via a dilution effect (Keesing et al., 2010). This effect is amplified in well‐mixed landscapes.

With respect to the durability of major resistance genes, the proportion of resistant fields had a U‐shaped effect in all deployment strategies (Figures 2a and 3a). This effect has already been described with mosaic strategies for the deployment of plant resistance (van den Bosch & Gilligan, 2003; Papaïx et al., 2018) or the application of pesticides (Bourget, Chaumont, & Sapoukhina, 2013). The higher durability at high cropping ratios is attributed to the large reduction in pathogen population size, resulting in a low probability of appearance of mutants (see the positive effect of cropping ratio on the time to first appearance of mutants in Supporting information Figure S13A). At small cropping ratios, high durability can be explained by the low probability that a mutant pathogen will successfully disperse to a resistant field (see the negative effect of cropping ratios, when below 50%, on the time to the first infection of a resistant host in Supporting information Figure S13B).

In contrast to its effect on epidemiological efficiency, spatial aggregation had a positive effect on resistance durability (Figures 2b and 5d). This is attributed to how different levels of aggregation alter the interface between resistant and susceptible components in an agricultural landscape (Papaïx et al., 2018). When this interface is small (i.e., there is a high level of aggregation), resistant cultivars are less exposed to potential mutant pathogens emerging from susceptible fields. On the contrary, disease spread in susceptible fields is less efficiently mitigated. It is noteworthy that we based our simulations on a landscape completely cultivated with host crops, an initial contamination of every susceptible field and an isotropic dispersal of the pathogen. Alternative scenarios should be more conducive to pathogen extinctions and would likely lead to an even greater influence of spatial aggregation.

#### 4.2.2 Impact of relative cropping ratios and relative aggregation

Within the different deployment options, we simulated different relative proportions and relative spatial/temporal aggregation of the resistance types. In many of our simulations, mosaics and especially mixtures resulted in the breakdown of only one major gene (Figure 2c) when resistant cultivars were deployed in unbalanced proportions (Figure 3c). More precisely, when two major resistance genes were deployed in uneven proportions, the durability of the gene in minority was increased to the detriment of the one in majority. Protection of the resistant cultivar in minority was likely due to specialization of the pathogen on the major cultivar. This conclusion, analogous to using refuge zones to influence pest evolutionary trajectories (Alstad & Andow, 1995), has interesting implications for agricultural systems where high‐value cultivars may be grown at a small scale in the neighbourhood of broadly grown standard cultivars.

In rotations, the length of the rotation had only a small impact on model outputs. As rust pathogens are biotrophs (i.e., they cannot survive in the absence of the host), and alternate hosts are absent in most of the large grain production areas, we simulated severe bottlenecks between seasons and considered that the end of a cropping season influenced the beginning of the next season only. However, as mentioned before, different results could be obtained with pathogens showing different life histories, such as those whose survival on stubbles or alternate hosts allows secondary infections for several years.

### 4.3 Pathogen mutation probability and infectivity costs have major effects on resistance durability

In an investigation of the durability of pyramided genes, Fabre, Bruchou, Palloix, and Moury (2009) found a strong effect of genetic mutation rate, the number of required genetic mutations, their nature (transition or transversion) and the associated fitness costs. In our study, we focused on phenotypic changes and integrated the first three of these variables into a mutation probability. This mutation probability and the associated cost of infectivity (which have been poorly characterized quantitatively and may be highly variable; Laine & Barrès, 2013) had a large influence on evolutionary outcomes for the simulated deployment strategies (Figure 2). It is not surprising that resistance durability was higher when the mutation probability was low and the cost of infectivity was high. These effects are especially strong with respect to the time to appearance and establishment of a superpathogen (Supporting information Figure S6), which corresponds to the durability of a pyramiding strategy. Pyramids of major resistance genes may therefore not be the best strategy when the target pathogen has a high probability of mutating towards infectivity (especially when there are only weak associated fitness costs).

In addition, our simulations highlight the synergistic interaction between the cost of infectivity and cropping ratios on the time to establishment of a superpathogen (Supporting information Figure S6) and the mid‐ and long‐term control of the disease (Supporting information Figures S8 and S9). This corroborates the results obtained by Fabre, Rousseau, Mailleret, and Moury (2012), suggesting that the optimal cropping ratio increases with increasing fitness costs. Overall, these results indicate that the harder it is for a pathogen to overcome a resistance gene, the more this resistance source can be cultivated in the landscape.

### 4.4 Conclusions and next challenges

In this study, we compared the main categories of resistance deployment: mosaics, mixtures, rotations, pyramiding and a variety of options, using a single ecoevolutionary framework. In line with the principles of integrated pest management and the illusory “one‐size‐fits‐all” pest control method (Barzman et al., 2015), none of the strategies we considered could be considered as a ‘‘universal optimum.” Indeed, as previously demonstrated for mosaics (van den Bosch & Gilligan, 2003; Papaïx et al., 2018), the optimal strategy depends on the objective of a given stakeholder group (e.g., breeders, growers, risk managers). Extended cultivar durability, prevention of superpathogen emergence, protection of susceptible crops or minimization of disease levels during growing seasons are all possible management targets that may not always be compatible and may require different strategies. Nevertheless, in the context of cereal resistance to rust fungi, given our model assumptions, we conclude that pyramiding is the strategy less likely to breakdown, but should that occur, the consequences may be drastic. On the contrary, although more likely to be overcome, alternative strategies better mitigate epidemic losses in the event of the breakdown of some or all sources of resistance.

Our results emphasize the impact of landscape organization on both epidemiological and evolutionary outcomes, but also show how the effectiveness of different strategies can be further modified by factors related to pathogen evolutionary ability. It is interesting that these factors (pathogen mutation probability and fitness cost of adaptation) may be influenced by the choice of the resistance source, as suggested by empirical evidence that major resistance genes acting with distinct mechanisms are associated with different rates of pathogen adaptation (Djian‐Caporalino et al., 2014; Mundt, 2018). Based on our results, and not surprisingly, resistance genes associated with small rates of pathogen adaptation (requiring several and costly genetic mutations to be overcome) must be favoured for deployment in the field.

Our conclusions may hold for a wide range of wind‐dispersed, biotrophic foliar pathogens, such as rusts of cereal crops, but could considerably differ with pathosystems showing contrasted life histories. Therefore, our next challenge will be to apply this modelling framework to other pathosystems associated with different dispersal and postharvest survival abilities and mode of reproduction. Different outcomes may be found, as parameters contributing to epidemic spread have been found to significantly impact both the resistance durability (Bourget et al., 2013) and epidemiological efficiency (Djidjou‐Demasse et al., 2017; Ohtsuki & Sasaki, 2006; Suzuki & Sasaki, 2011) of different deployment strategies. It will also be of interest to explore more complex strategies that combine several types of deployment and both spatial and temporal genetic host diversity. As shown by previous studies, we expect some combinations to favour resistance durability, such as rotations and mosaics (Fabre et al., 2015; Lof et al., 2017), or, as suggested before, rotations and pyramids. On the other hand, cultivating pyramids together with cultivars carrying only single resistance genes has the opposite effect (Bourget et al., 2013; Lof et al., 2017). We hope that the modelling ecoevolutionary framework presented here will provide a solid foundation for such future and interesting investigations.

## DATA ARCHIVING STATEMENT

Raw data for this study are available in Supporting Information. The model is available in the R package *landsepi* (Rimbaud, Papaïx, Rey et al., 2018).
