# Supplementary material for: Mosaics, mixtures, rotations or pyramiding: What is the optimal strategy to deploy major gene resistance?
Source: Evol Appl. 2018 Sep 17;11(10):1791–810. doi: 10.1111/eva.12681 (PMC6231482; doi:10.1111/eva.12681)
Supplement: Supplementary file 5 [file EVA-11-1791-s005.pdf]

Supporting Information

Assessing the durability and efficiency of landscape-based strategies to deploy plant resistance to pathogens

The following supporting information is available for this article:

Fig. S1. Spatial allocation of cultivars.

Fig. S2. Effect of landscape structure on model outputs.

Fig. S3. Sensitivity analyses of the durability of the 1st major resistance gene.

Fig. S4. Sensitivity analyses of the durability of the 2nd major resistance gene.

Fig S5. Durability of the 2nd major resistance gene.

Fig. S6. Time to establishment of the super-pathogen in resistant host populations.

Fig. S7. Sensitivity analyses of short-term epidemiological control.

Fig. S8. Sensitivity analyses of epidemiological control during the transitory period.

Fig. S9. Sensitivity analyses of long-term epidemiological control.

Fig. S10. Sensitivity analyses of global epidemiological control.

Fig. S11. Principal component analysis: projection of model outputs.

Fig. S12. Principal component analysis: dots associated with high mutation probabilities.

Fig. S13. Effect of the proportion of resistant fields on times to first mutation and first infection.

Table S1. Goodness-of-fit of polynomial regressions.

Videos. Dynamics of diseased hosts in the landscape following the deployment of a resistant cultivar (φ_1=5/6; α_1=medium) carrying two major resistance genes (φ_2=1/2; α_2=medium; θ=0.75) in a set of 48-year simulations (one year per second). The left panel shows the area under disease progress curve (AUDPC). The vertical blue lines marks the time to breakdown of the major resistance genes. The right panel shows the spatiotemporal dynamics of the epidemic in the simulated landscape.

- Video S1. Deployment in a mosaic strategy at low mutation probability ($\tau=10^{-7}$).
- Video S2. Deployment in a mixture strategy at low mutation probability ($\tau=10^{-7}$).
- Video S3. Deployment in a rotation strategy at low mutation probability ($\tau=10^{-7}$).
- Video S4. Deployment in a pyramiding strategy at high mutation probability ($\tau=10^{-4}$).

Raw data.rds File containing raw data in binary RDS format. Use the function *readRDS*() to open in R. The table contains the following notations:

**Simulation plan**	
**strat**<br>- **MO2**<br>- **MI2**<br>- **RO2**<br>- **PY2**	Category of resistance deployment:<br>- mosaic<br>- mixture<br>- rotation<br>- pyramiding
**taumut**	τ
**idLAN**	Index of landscape structure
**propSR**	φ_1
**isolSR**	α_1
**propRR**	φ_2
**isolRR**	α_2
**costInfect**	θ
**Model outputs**	
**extinction**	Whether or not the pathogen is extinct before the end of the simulation period
**audpcST**	Short-term control, computed on the susceptible cultivar from the beginning of the simulation until one of the major resistance gene is overcome
**audpcTP**	Control during the transitory period when only one major resistance gene is overcome, computed on the susceptible cultivar
**audpcLT**	Long-term control, computed on the whole landscape from the time both major resistance genes are overcome until the end of the simulation run
**audpcTOT**	Global control, computed on the whole landscape across the whole simulation run
**audpc_SC**	Disease severity on the susceptible cultivar
**audpc_RC1**	Disease severity on resistant cultivar 1, carrying major resistance gene 1
**audpc_RC2**	Disease severity on resistant cultivar 2, carrying major resistance gene 2
**mut1**	First appearance of a mutant carrying infectivity gene 1
**mut2**	First appearance of a mutant carrying infectivity gene 2
**mut12**	First appearance of the super-pathogen
**inf1**	First infection of a resistant host by a mutant carrying infectivity gene 1
**inf2**	First infection of a resistant host by a mutant carrying infectivity gene 2
**inf12**	First infection of a resistant host by the super-pathogen
**dur1**	Broader establishment of a mutant carrying infectivity gene 1 in the resistant host population
**dur2**	Broader establishment of a mutant carrying infectivity gene 2 in the resistant host population
**dur12**	Broader establishment of the super-pathogen in the resistant host population

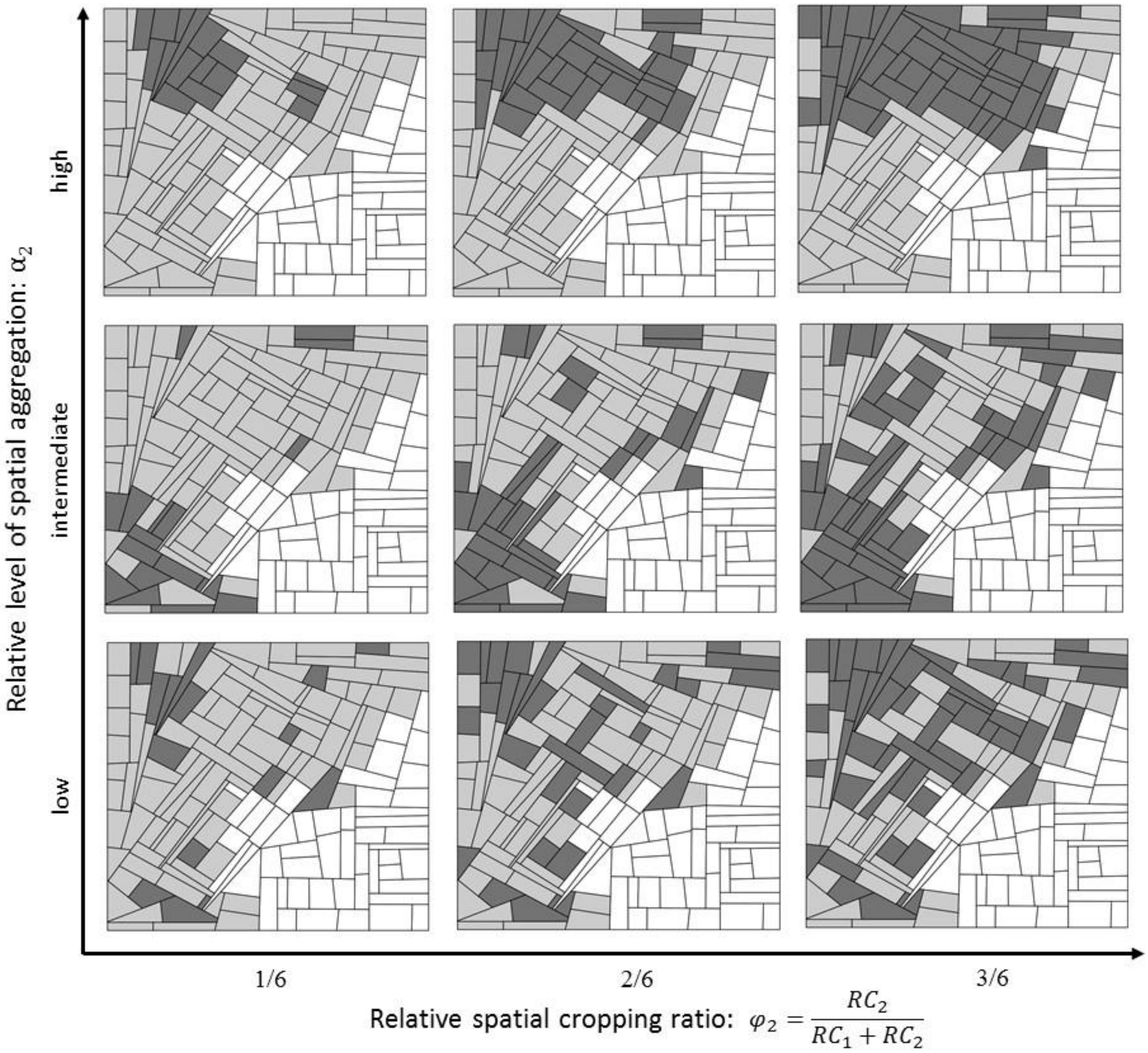

Fig. S1. Spatial allocation of cultivars. A landscape structure is generated using T-tessellations. Fields are then allocated with a susceptible (white) and two resistant cultivars (RC_1, light grey, and RC_2, dark grey), with controlled relative proportions of the surface coverage (φ_2, horizontal axis) and level of spatial aggregation (α_2, vertical axis) of RC_1 and RC_2. The total proportion of resistant fields is φ_1=2/3 and their level of aggregation is α_1=high.

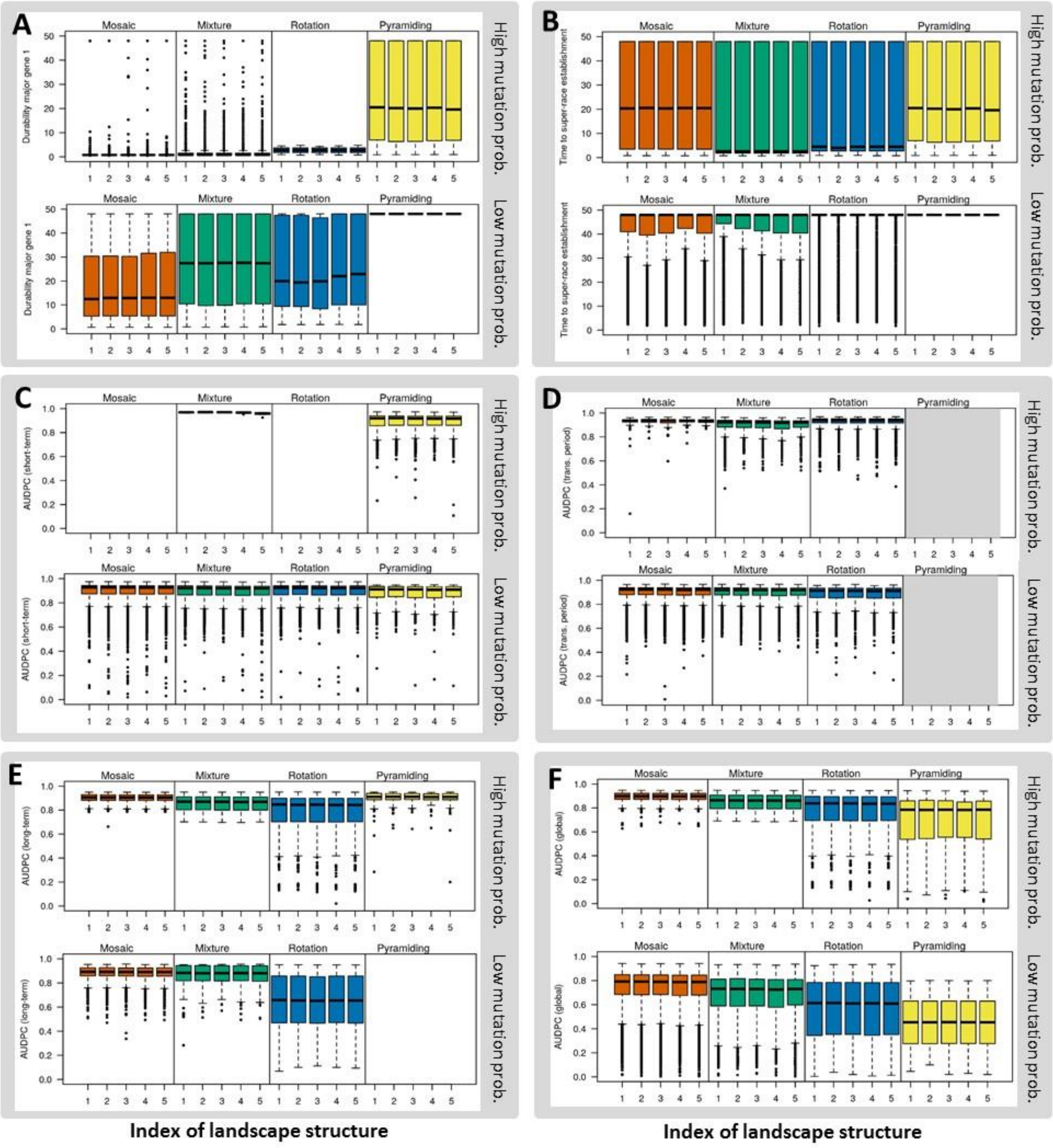

Fig. S2. Effect of landscape structure on model outputs. Five different landscapes structures (spatial arrangement of field boundaries) of about 150 fields were used in this study. They were generated using a T-tesselation algorithm. They never had a significant impact on evolutionary and epidemiological model outputs: durability of the 1st major resistance gene (Dur_1, in years, A), time to establishment of the super-pathogen (Dur_{12}, in years, B), short-term disease control (AUDPC_{ST}, C), disease control in the transitory period (AUDPC_{TP}, D), long-term disease control (AUDPC_{LT}, E), and global control (AUDPC_{TOT}, F). Note that in pyramids $\mathrm{Dur}_1=\mathrm{Dur}_2=\mathrm{Dur}_{12}$.

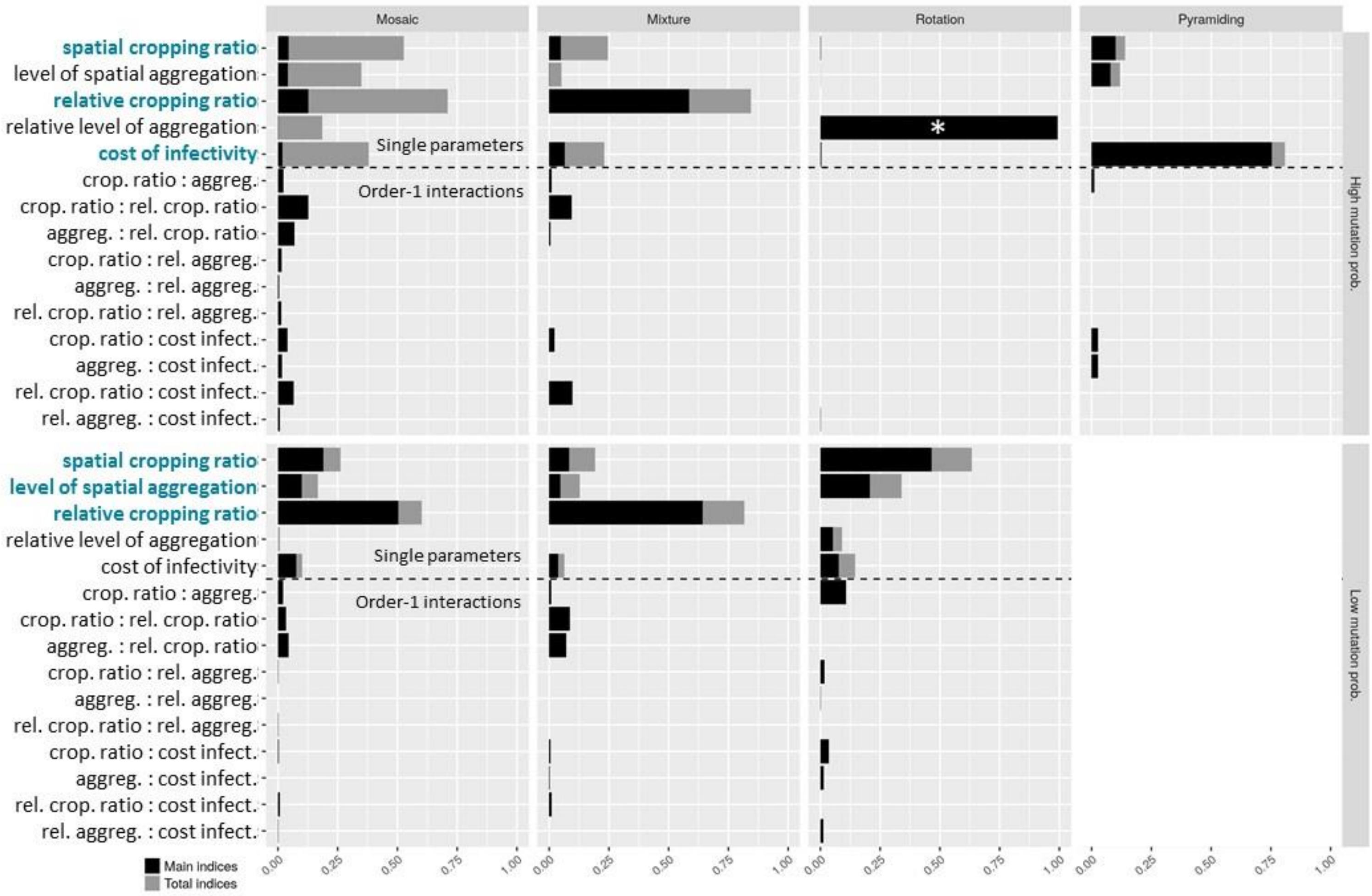

Fig. S3. Sensitivity analyses of the durability of the 1st major resistance gene. Sensitivity indices were computed using Legendre polynomials within a Poisson generalised linear model. The main sensitivity index of a given input parameter (black) reflects its main influence on resistance durability (Dur_1), at high ($\tau=10^{-4}$) and low ($\tau=10^{-7}$) mutation probabilities, whereas the total index (grey) includes its interactions with other parameters. Order-2 interactions have not been represented (maximal index of 0.09). *: The strong influence of the relative level of temporal aggregation in rotations is an artefact of the model owing to the computation of resistance durability from the beginning of the simulation. Rotations were simulated by starting with the second resistant cultivar (RC_2), so parameter α_2 directly impacted the year of deployment of the first resistant cultivar (RC_1, carrying the 1st major resistance gene). Note that pyramids were never overcome at low mutation probabilities.

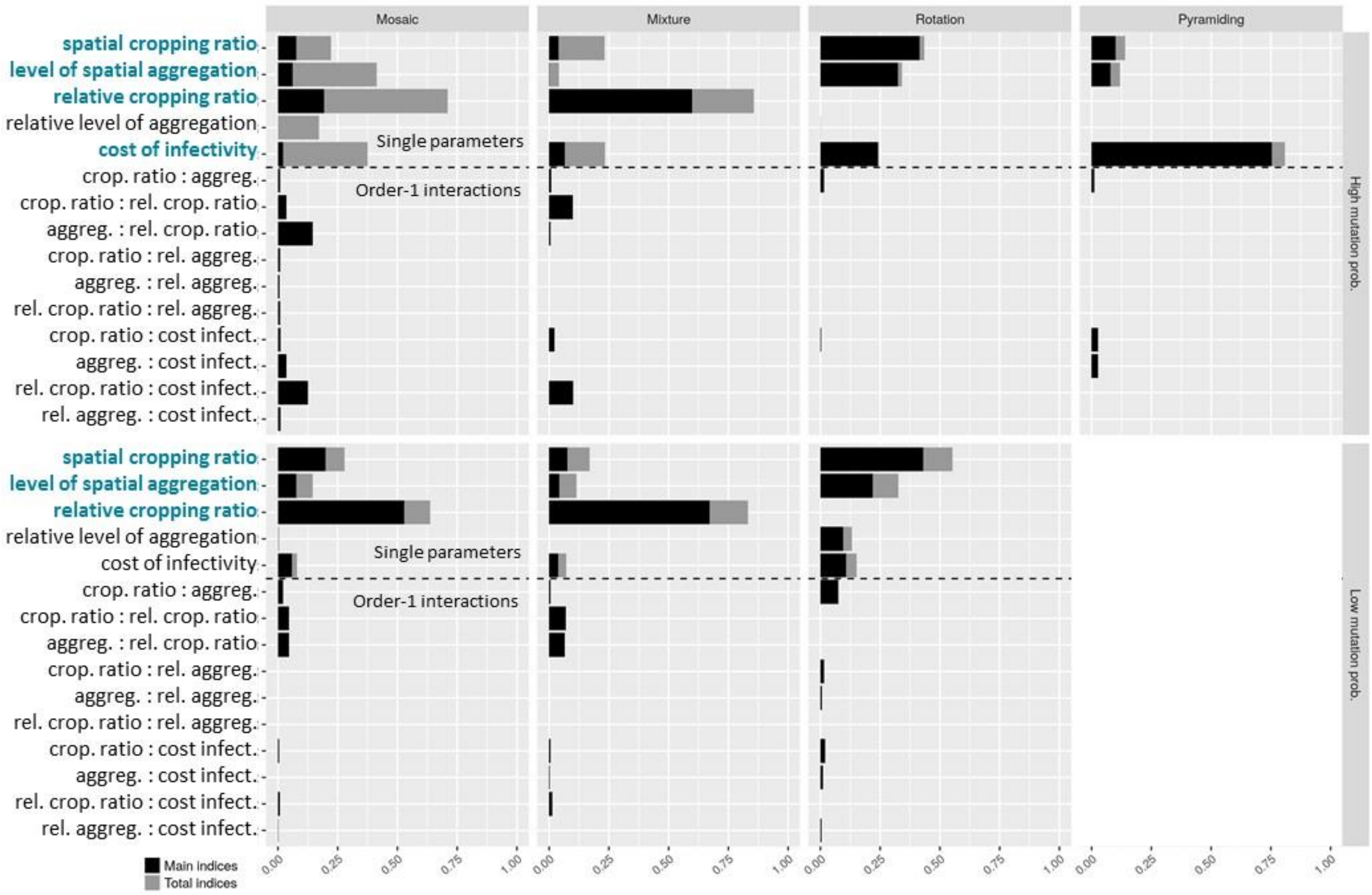

Fig. S4. Sensitivity analyses of the durability of the 2nd major resistance gene. Sensitivity indices have been computed using Legendre polynomials within a Poisson generalised linear model. The main sensitivity index of a given input parameter (black) reflects its main influence on resistance durability (Dur_2), at high ($\tau=10^{-4}$) and low ($\tau=10^{-7}$) mutation probabilities, whereas the total index (grey) includes its interactions with other parameters. Order-2 interactions have not been represented (maximal index of 0.11). Note that pyramids were never overcome at low mutation probabilities.

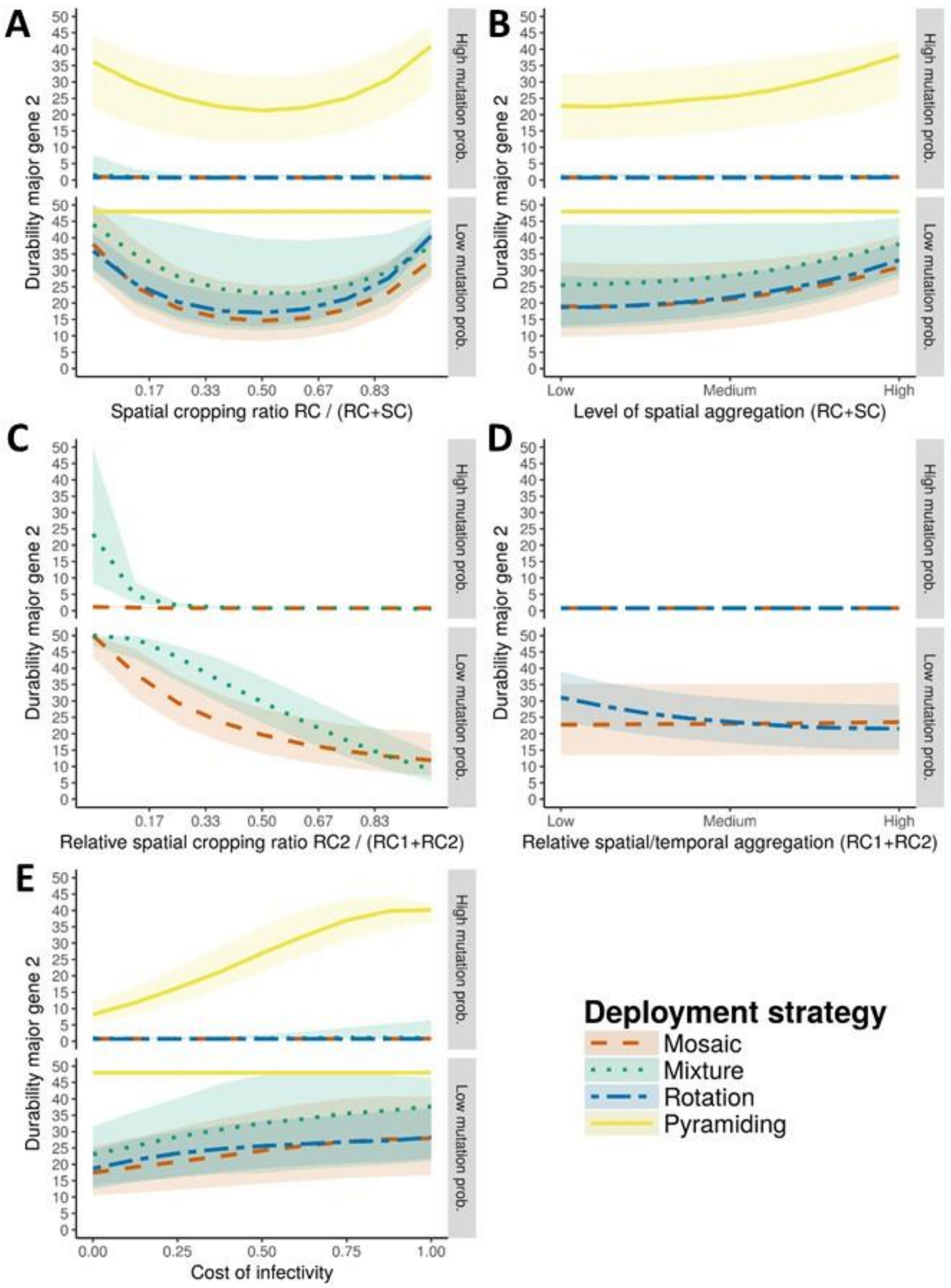

Fig S5. Durability of the 2nd major resistance gene. Durability (in years) of the second major resistance gene (Dur_2) at high ($\tau=10^{-4}$) and low ($\tau=10^{-7}$) mutation probabilities. Panels show the effect of the proportion of fields where resistance is deployed (A), their level of spatial aggregation (B), the relative proportion of the second major gene (C), its relative level of spatial (for mosaics) or temporal (for rotations) aggregation (D), and the fitness cost associated with pathogen infectivity (E). Curves represent the median prediction using 3rd degree Legendre polynomials including interactions up to 2nd order within a Poisson generalised linear models; envelopes are delimited by the 1st and 3rd quartiles. SC: susceptible cultivar; RC: resistant cultivars, including the first (RC_1) and the second (RC_2) resistance gene. Note that when a major resistance gene remains effective during the whole simulation run, its durability is set at 48 years, and that in pyramids, $\mathrm{Dur}_1=\mathrm{Dur}_2=\mathrm{Dur}_{12}$.

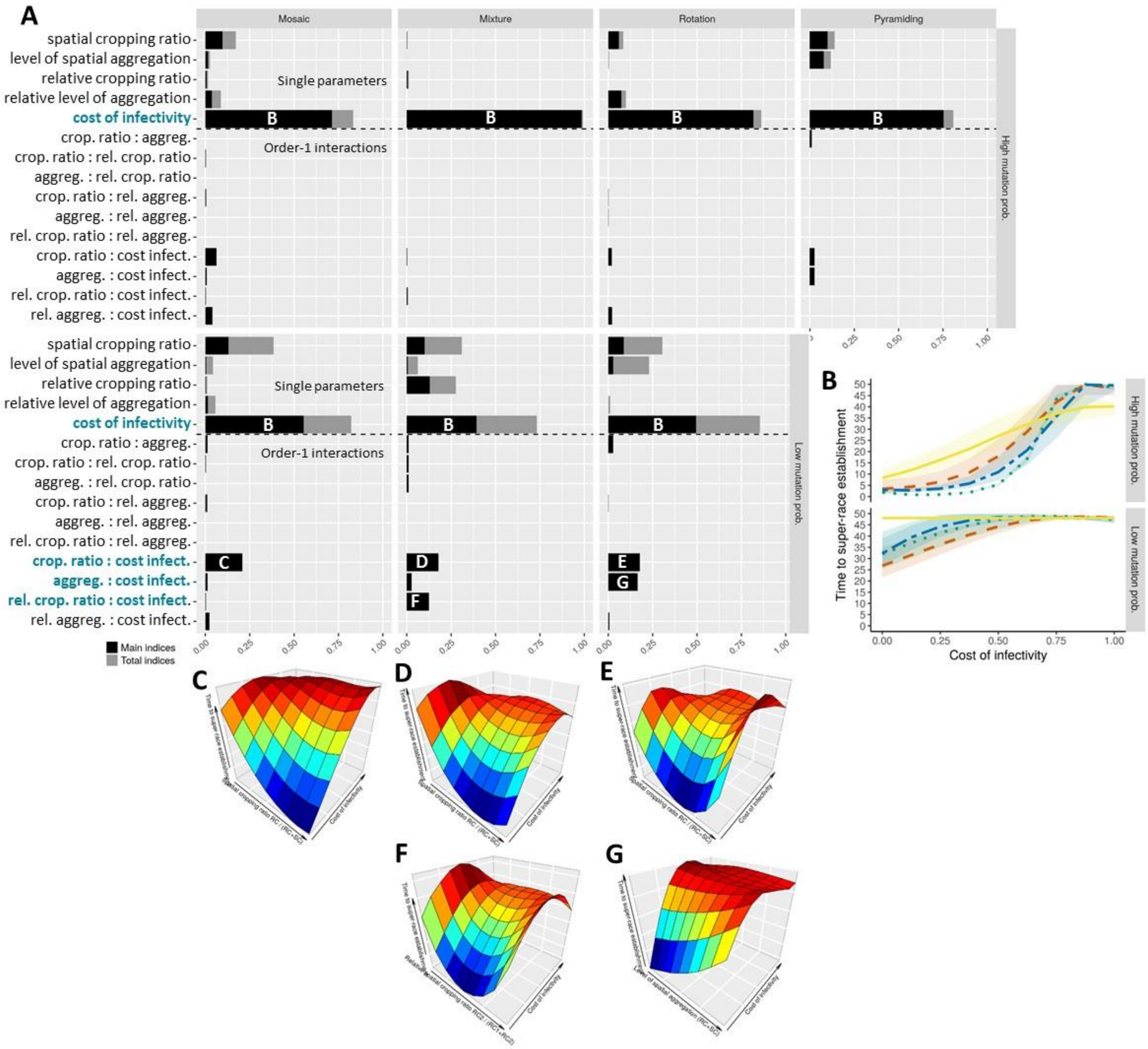

Fig. S6. Time to establishment of the super-pathogen in resistant host populations. Sensitivity analyses based on Legendre polynomials within a Poisson generalised linear model enabled the computation of sensitivity indices of input parameters on the time to establishment of the super-pathogen (Dur_{12}), at high ($\tau=10^{-4}$) and low ($\tau=10^{-7}$) mutation probabilities (A). The main sensitivity index of a given input parameter (black) reflects its main influence on the model output, whereas the total index (grey) includes its interactions with other parameters. Order-2 interactions have not been represented because they were negligible (maximal index of 0.01). Polynomial regressions were used to predict the time to super-pathogen establishment (in years) for different values of cost of infectivity (B), and at low mutation probability for different combinations of cost of infectivity and the proportion of fields where resistance is deployed in mosaics (C), mixtures (D) and rotations (E), or cost of infectivity and relative proportion of the second major resistance gene in mixtures (F), and finally cost of infectivity and level of spatial aggregation of resistant fields in rotations (G). In (A), white letters indicate figure panels of predictions associated with sensitivity indices. In (B), solid lines represent the median and envelopes are delimited by the 1st and 3rd quartiles. Pyramids were never overcome at low mutation probabilities.

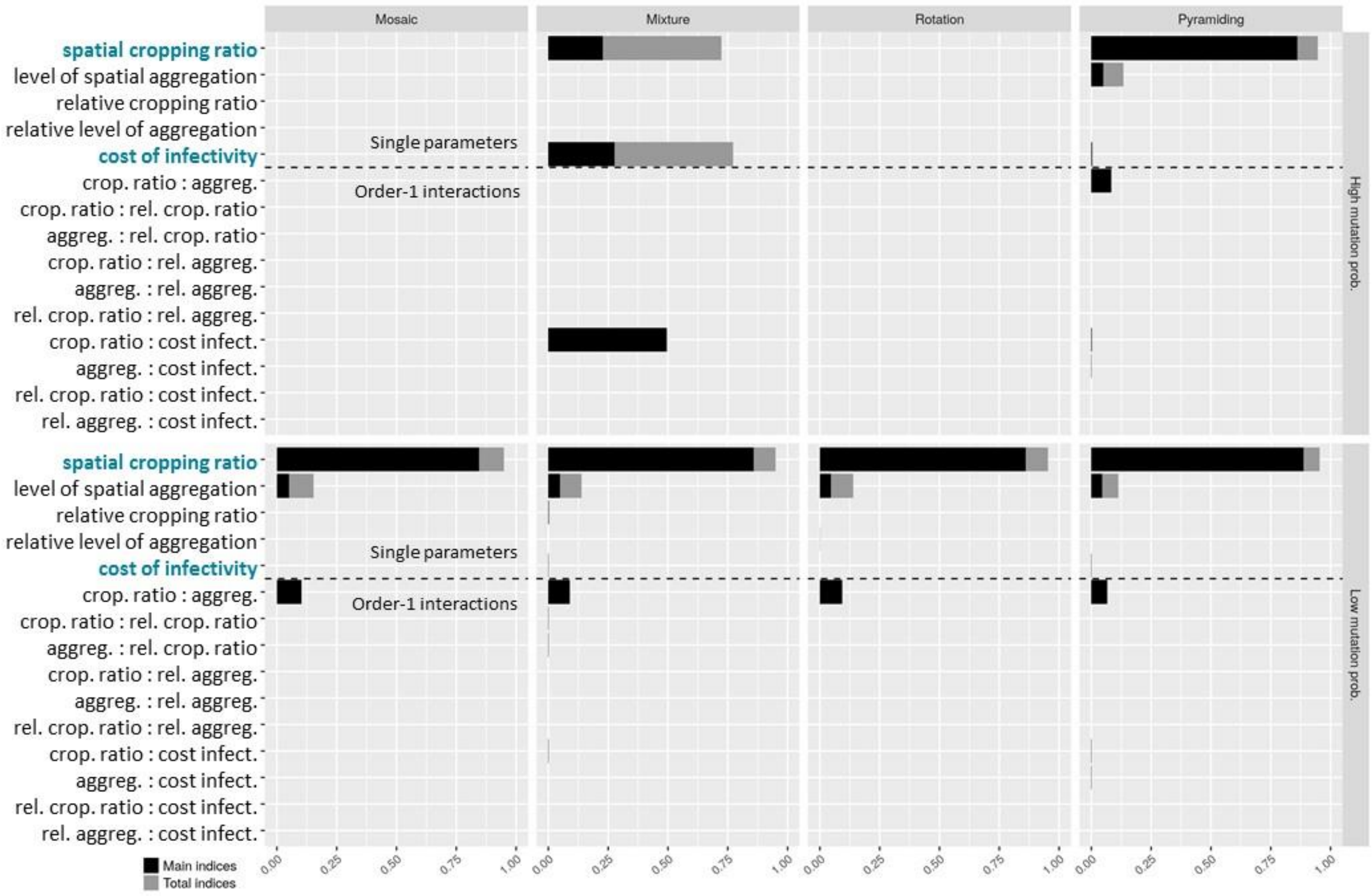

Fig. S7. Sensitivity analyses of short-term epidemiological control. Sensitivity indices were computed using Legendre polynomials. The main sensitivity index of a given input parameter (black) reflects its main influence on disease control when all resistances are still effective ($AUDPC_{ST}$), at high ($\tau=10^{-4}$) and low ($\tau=10^{-7}$) mutation probabilities, whereas the total index (red) includes its interactions with other parameters. Order-2 interactions have not been represented because they were negligible (maximal index of 2.10^{-4}). Note that mosaics, mixtures and rotations were almost always overcome in less than one year. In these cases $AUDPC_{ST}$ could not be properly computed.

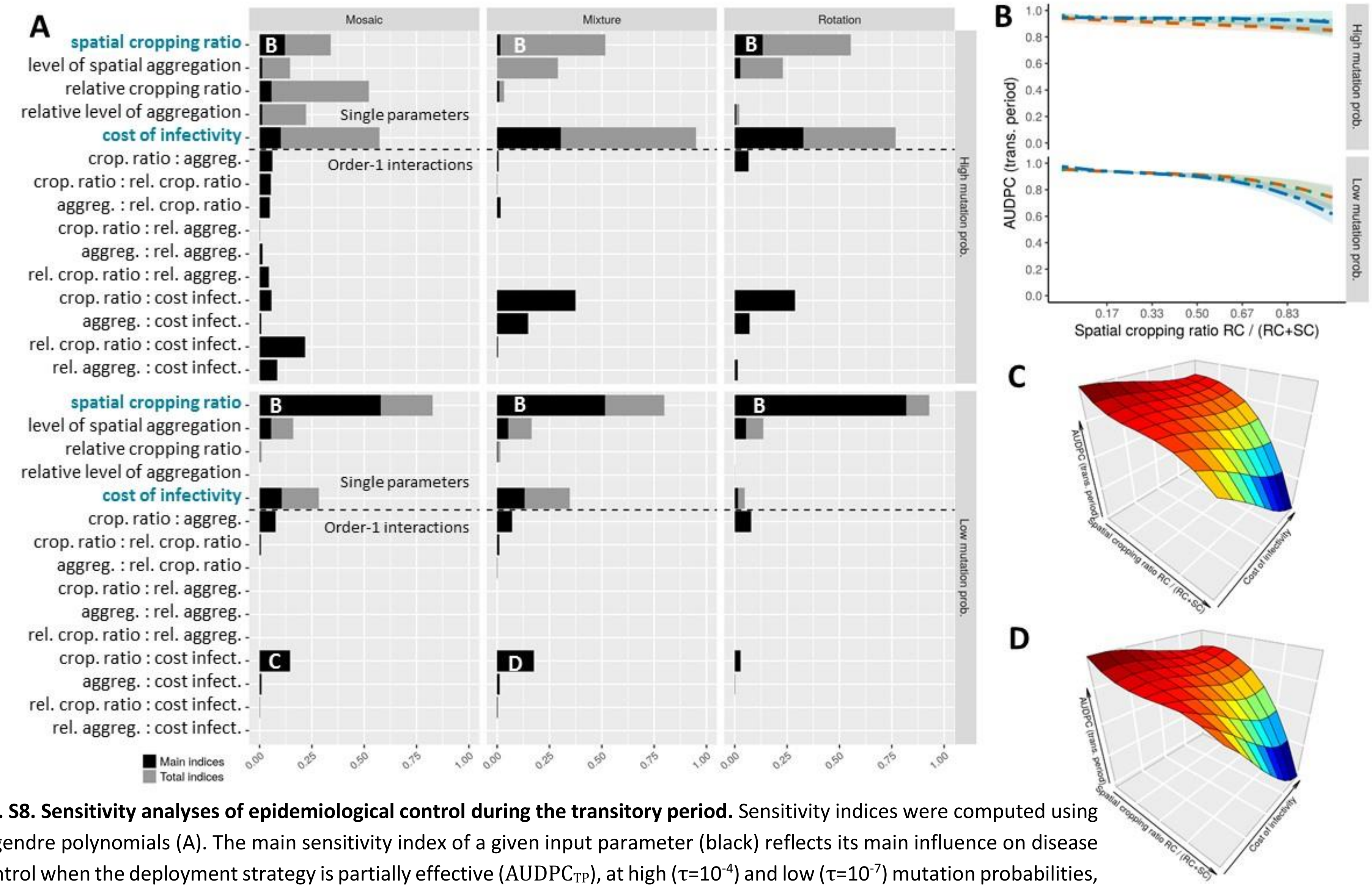

Fig. S8. Sensitivity analyses of epidemiological control during the transitory period. Sensitivity indices were computed using Legendre polynomials (A). The main sensitivity index of a given input parameter (black) reflects its main influence on disease control when the deployment strategy is partially effective ($AUDPC_{TP}$), at high ($\tau=10^{-4}$) and low ($\tau=10^{-7}$) mutation probabilities, whereas the total index (red) includes its interactions with other parameters. Order-2 interactions have not been represented (maximal index of 0.11). Polynomial regressions were used to predict the model output for different values of proportion of resistant fields in the landscape (B), or different combinations of the proportion of resistant fields and cost of infectivity (C-D). In (A), white letters indicate the figure panel of predictions associated with sensitivity indices. In (B), solid lines represent the median and envelopes are delimited by the 1st and 3rd quartiles. Note that the transitory period cannot exist in pyramids.

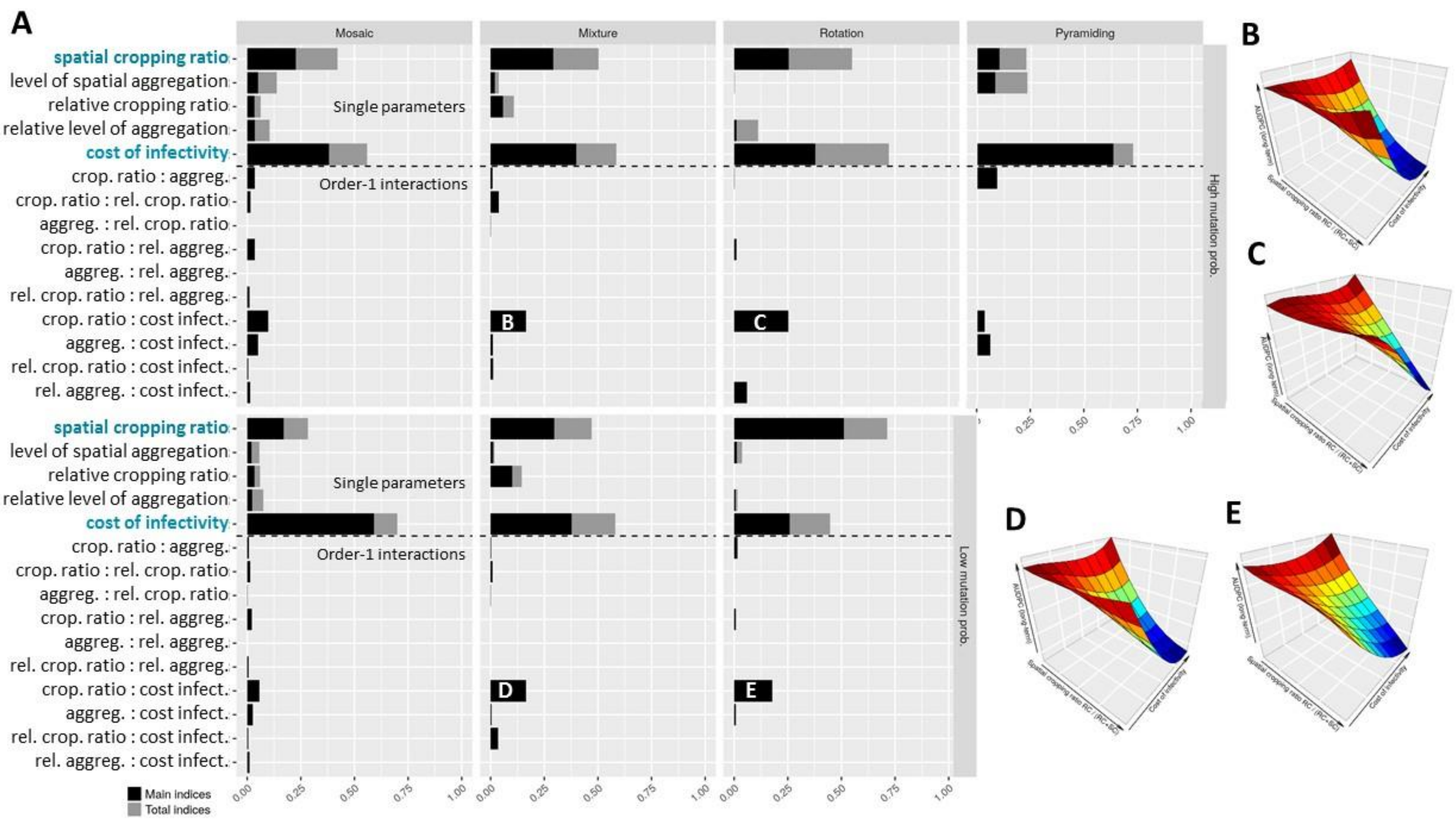

Fig. S9. Sensitivity analyses of long-term epidemiological control. Sensitivity indices were computed using Legendre polynomials. The main sensitivity index of a given input parameter (black) reflects its main influence on the long-term control when all resistance have been overcome ($\mathrm{AUDPC_{LT}}$), at high ($\tau=10^{-4}$) and low ($\tau=10^{-7}$) mutation probabilities, whereas the total index (grey) includes its interactions with other parameters. Order-2 interactions have not been represented because they were negligible (maximal index of 0.03). The polynomial regressions were used to predict the model output for different combinations of the proportion of resistant fields and cost of infectivity (B-E). In (A), white letters indicate the figure panel of predictions associated with sensitivity indices. Note that pyramids were never overcome at low mutation probabilities.

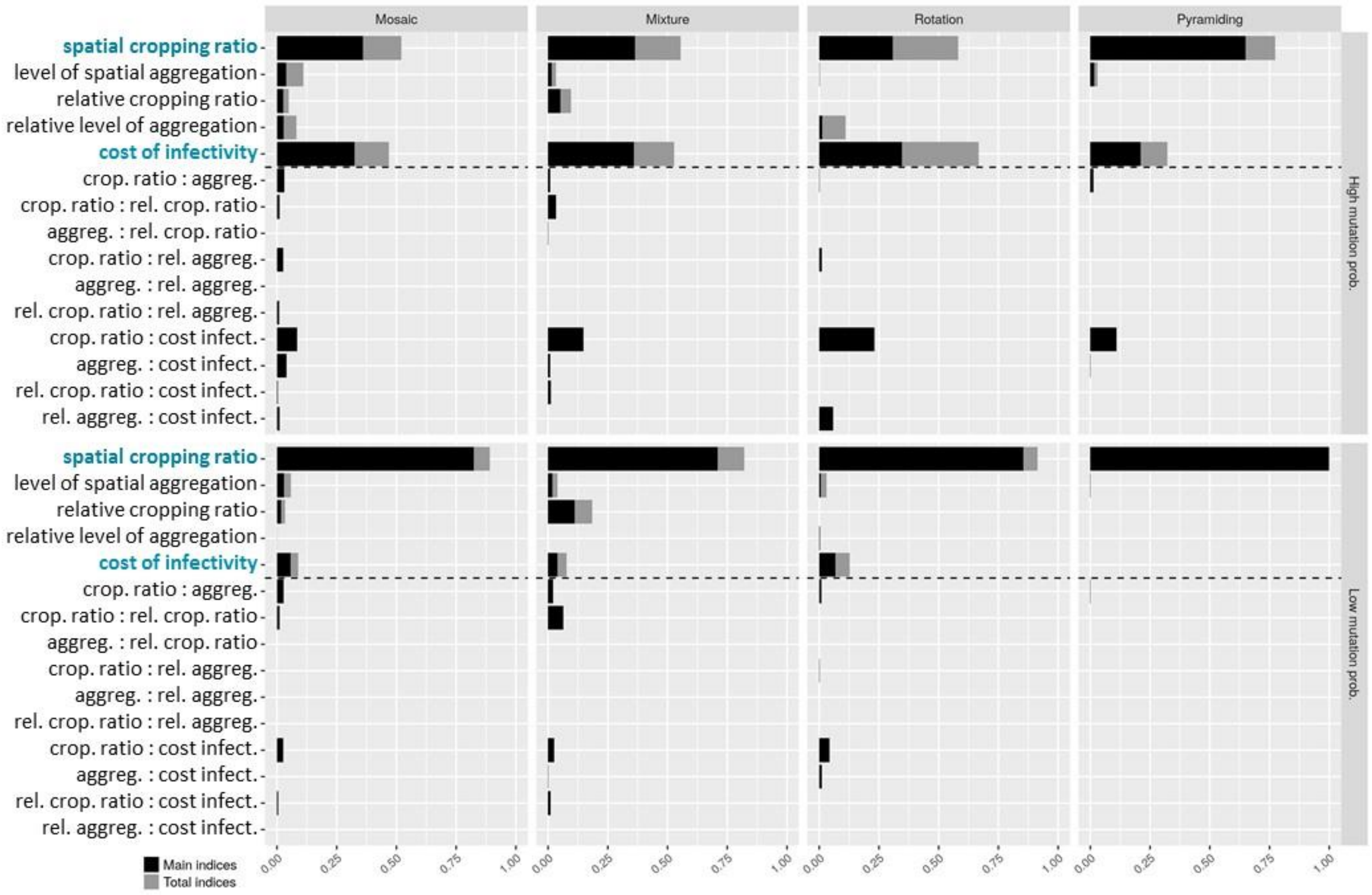

Fig. S10. Sensitivity analyses of global epidemiological control. Sensitivity indices were computed using Legendre polynomials. The main sensitivity index of a given input parameter (black) reflects its main influence on the overall epidemiological disease control ($\mathrm{AUDPC_{TOT}}$), at high ($\tau=10^{-4}$) and low ($\tau=10^{-7}$) mutation probabilities, whereas the total index (grey) includes its interactions with other parameters. Order-2 interactions have not been represented as they were negligible (maximal index of 0.03).

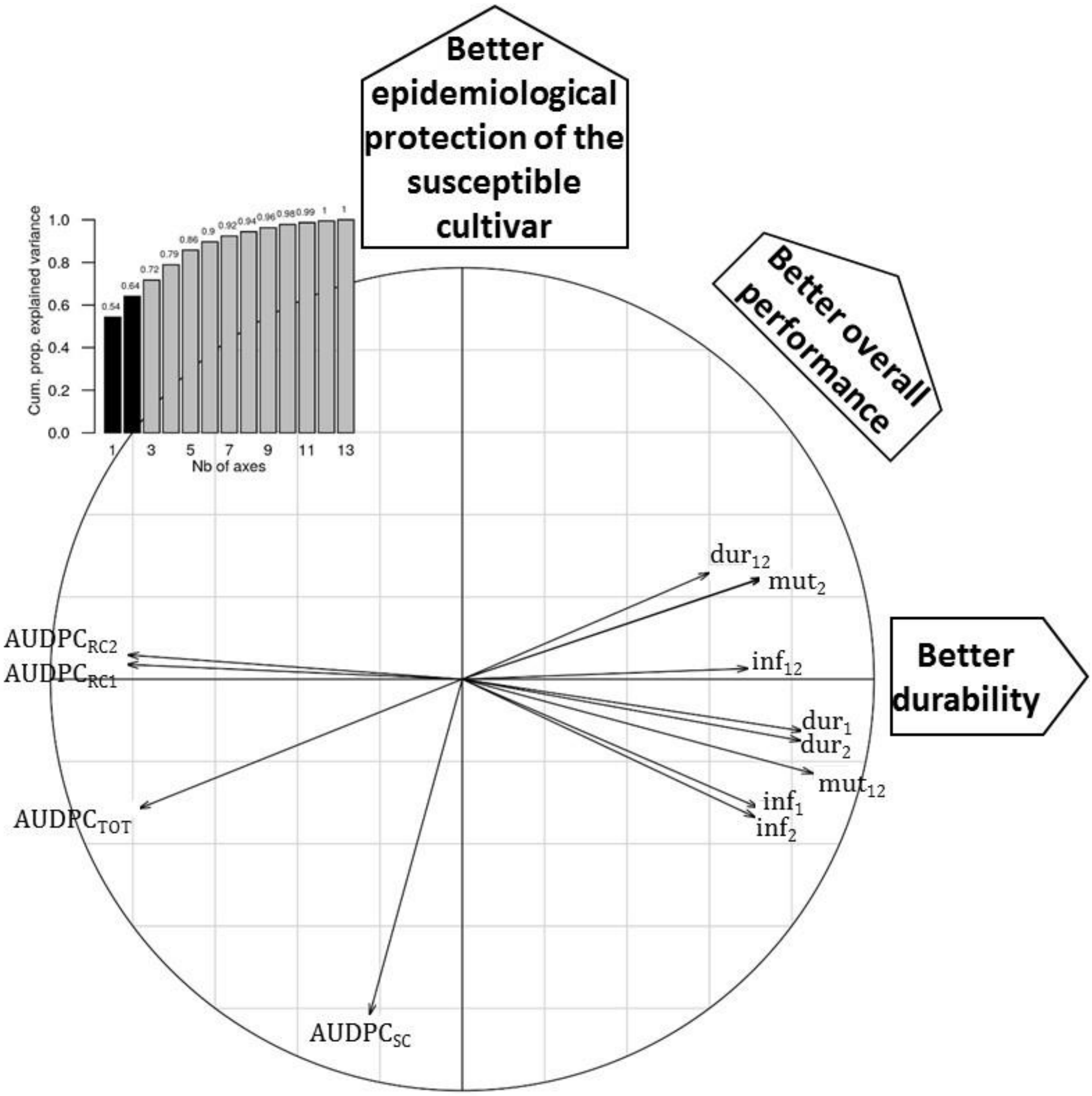

Fig. S11. Principal component analysis: projection of model outputs. Projection of evolutionary (Mut_1, Mut_2, Mut_{12}, Inf_1, Inf_2, Inf_{12}, Dur_1, Dur_2, Dur_{12}, see Table 3 for details) and epidemiological ($AUDPC_{SC}$, $AUDPC_{RC1}$, $AUDPC_{RC2}$, $AUDPC_{TOT}$) model outputs on the two main axes (total explained variance: 64%). Inset: cumulative proportion of variance explained by the axes of the PCA.

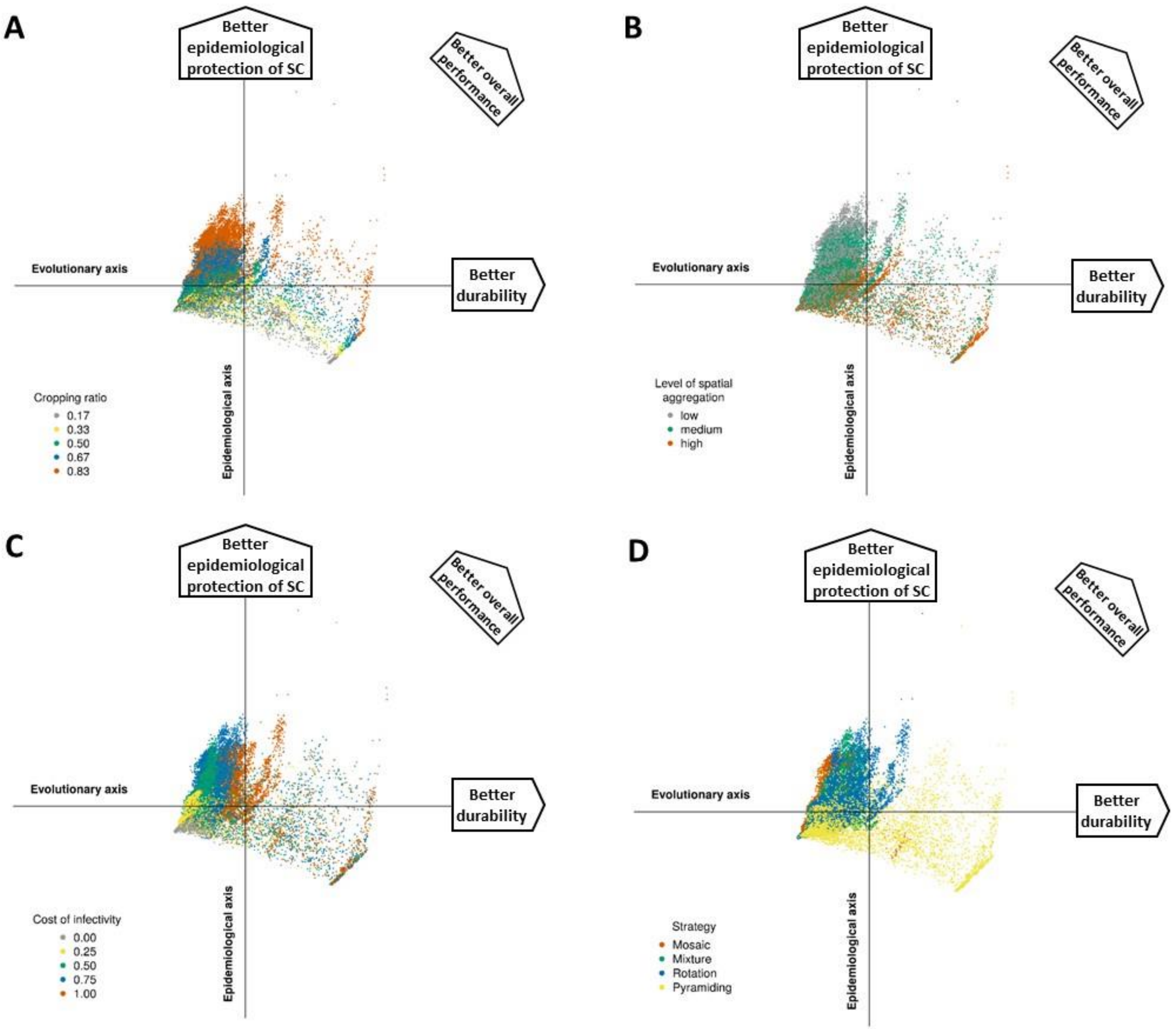

Fig. S12. Principal component analysis: dots associated with high mutation probabilities (τ=10^{-4}). Projection of the simulation results on the two main axes (total explained variance: 64%), with colour codes reflecting: (A) the proportion of fields where resistance was deployed; (B) their level of spatial aggregation; (C) the fitness cost associated with pathogen infectivity, and; (D) the category of the deployment strategy.

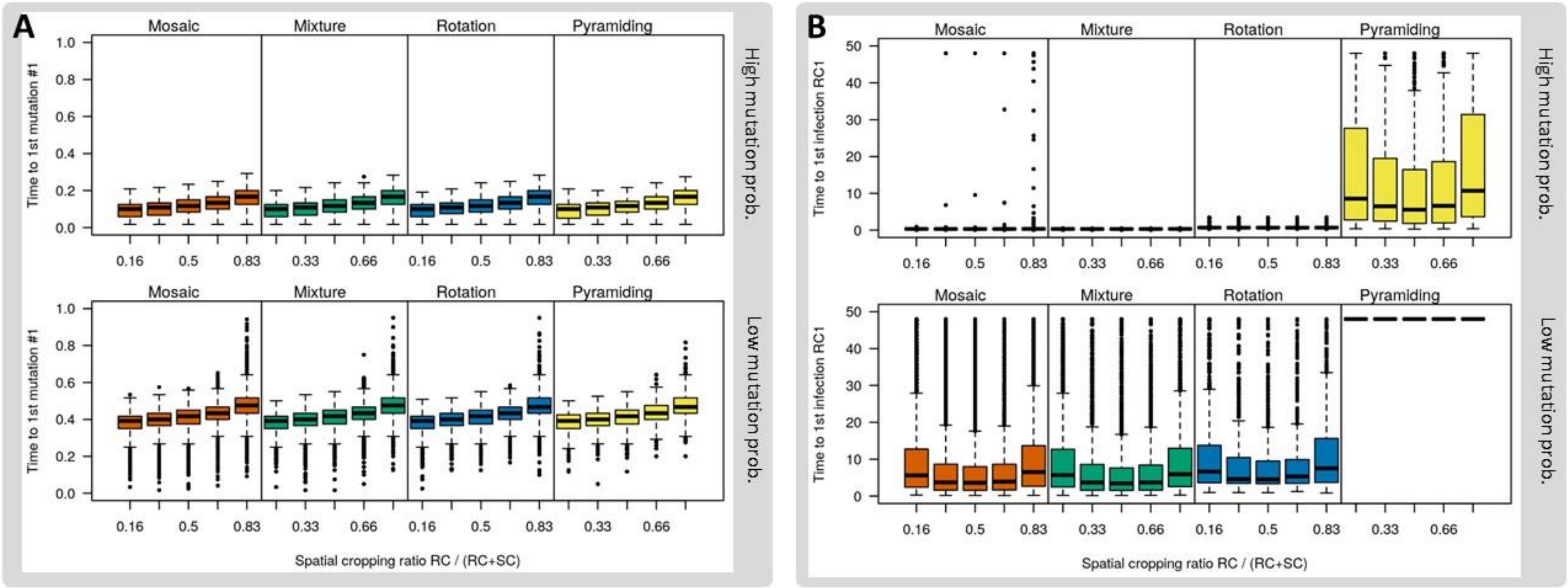

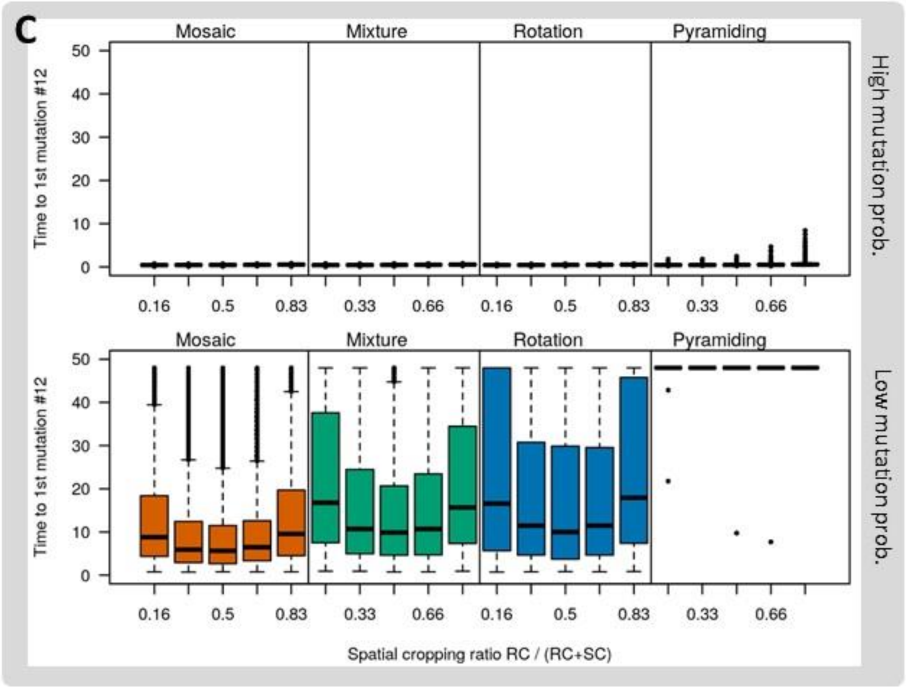

Fig. S13. Effect of the proportion of resistant fields on times to first mutation and first infection. Time to first appearance of mutants able to overcome major resistance gene 1 (Mut_1, A), first infection of resistant hosts (RC_1) by these mutants (Inf_1, B), and time to first appearance of super-pathogens (able to overcome both major resistance genes 1 and 2, Mut_{12}, C), at high ($\tau=10^{-4}$) and low ($\tau=10^{-7}$) mutation probabilities. Vertical axes are expressed in years. Note that for the pyramiding strategy, $\mathrm{Inf}_1= \mathrm{Inf}_2=\mathrm{Inf}_{12}$.

Table S1. Goodness-of-fit of polynomial regressions. Evolutionary outputs were fitted by 3rd degree Legendre polynomials including interactions up to 2nd order, and restricted to polynomial terms of up to degree 3, in Poisson generalised models. These regressions were assessed using explained deviance (D). Epidemiological outputs, based on AUDPC, were fitted by the same Legendre polynomial in linear models, and were assessed using the coefficient of determination (R^2).

Model output	Mutation prob.	Strategy	D or R^2
Durability of the 1st major resistance gene	High	Mosaics	0.34
		Mixtures	0.85
		Rotations	0.98
		Pyramiding	0.45
	Low	Mosaics	0.33
		Mixtures	0.46
		Rotations	0.23
		Pyramiding	-
Durability of the 2nd major resistance gene	High	Mosaics	0.32
		Mixtures	0.86
		Rotations	0.50
		Pyramiding	0.45
	Low	Mosaics	0.35
		Mixtures	0.46
		Rotations	0.22
		Pyramiding	-
Time to establishment of the super-pathogen	High	Mosaics	0.75
		Mixtures	0.94
		Rotations	0.88
		Pyramiding	0.45
	Low	Mosaics	0.38
		Mixtures	0.39
		Rotations	0.43
		Pyramiding	-
$AUDPC_{ST}$	High	Mosaics	-
		Mixtures	0.21
		Rotations	-
		Pyramiding	0.74
	Low	Mosaics	0.69
		Mixtures	0.73
		Rotations	0.71
		Pyramiding	0.81
$AUDPC_{TP}$	High	Mosaics	0.21
		Mixtures	0.78
		Rotations	0.79
		Pyramiding	

	Low	Mosaics	0.79
		Mixtures	0.83
		Rotations	0.80
		Pyramiding	-
**$AUDPC_{LT}$**	High	Mosaics	0.96
		Mixtures	0.98
		Rotations	0.97
		Pyramiding	0.50
	Low	Mosaics	0.71
		Mixtures	0.89
		Rotations	0.84
		Pyramiding	-
**$AUDPC_{TOT}$**	High	Mosaics	0.94
		Mixtures	0.98
		Rotations	0.97
		Pyramiding	0.63
	Low	Mosaics	0.57
		Mixtures	0.64
		Rotations	0.89
		Pyramiding	1.00
